# Multi-omic data integration improves the resolution of the molecular etiology of autism in a mouse model

**DOI:** 10.1038/s41380-026-03772-4

**Published:** 2026-07-25

**Authors:** Carolina D. Alberca, Kwangmoon Park, Ligia A. Papale, Andy Madrid, Phillip E. Bergmann, Sündüz Keleş, Reid S. Alisch

**Affiliations:** 1 Department of Neurological Surgery, University of Wisconsin School of Medicine and Public Health, 600 Highland Ave, Madison, WI 53792 USA; 2 Department of Statistics, University of Wisconsin, Medical Sciences Center, 1300 University Ave Room 1220, Madison, WI 53706 USA; 3 Department of Biostatistics and Medical Informatics, University of Wisconsin School of Medicine and Public Health, 600 Highland Ave, Madison, WI 53792 USA

**Keywords:** Genetics, Autism spectrum disorders

## Abstract

Autism spectrum disorder (ASD) is a multifactorial neurodevelopmental disorder with complex molecular etiology. Since genetic causes account for less than 50% of ASD cases, novel approaches are required to overcome the challenges of characterizing genes and molecular pathways linking risk alleles to phenotype. Here we use RNA-sequencing, 3-dimensional protein-centric chromatin conformation (Hi-ChIP), and whole genome DNA methylation sequencing approaches to investigate hippocampal tissue from an ASD mouse model (*Cntnap2* knockout [*Cntnap2* KO]) to determine if multi-omic data integration improves the resolution of key molecular pathways contributing to the complex ASD phenotype. Each -omic dataset individually identified disruptions in numerous genes and pathways, providing broad ASD-related insights, such as 1) 699 downregulated genes with an enrichment of neuronal ontological terms; 2) unique chromatin interactions in *Cntnap2* KO mice; and 3) that most differentially methylated genes (1220/1659) have a neuronal function. The multi-omic data integration reduced the heterogeneity and refined the data to 43 genes with links to ASD (*e.g*., *Zbtb18, Cttnbp2, Gabbr2*). A pathways analysis of these 43 genes identified one gene ontological term: regulation of neuronal synaptic plasticity. These findings are consistent with large scale gene expression studies of human ASD postmortem brain tissue, suggesting that multi-omic data integration can be used to achieve a greater resolution of the heterogeneous ASD molecular etiology.

## Introduction

Autism spectrum disorder (ASD) is a complex and heterogeneous neurodevelopmental condition encompassing a range of severity, including characteristics such as increased repetitive behaviors and deficits in sociability and communication [1]. The genetic etiology of ASD is also heterogeneous, as ~1000 genes have been implicated [2]; however, molecular pathways linking these risk loci to phenotype remain unclear. Large scale gene expression studies of ASD postmortem brain tissue, as well as the prenatal and postnatal developing brain, suggest alterations in molecular pathways contributing to synaptic and immune functions during brain development contribute to the ASD pathophysiology [3]. ASD postmortem brain tissue also exhibits DNA methylation differences in genes and pathways related to immune function and neuronal regulation [3]. Additionally, differences in histone acetylation, H3K27ac, an epigenetic mark of active chromatin, have been associated with genes involved in synaptic transmission and morphogenesis [4]. Major molecular contributors to neurodevelopment reside in non-coding regions of the genome (*e.g*., enhancers); thus, long-range chromatin interactions between enhancer and promoter regions of the genome are critical to identify the potential functional targets for neurodevelopmental disorders [5].

CNTNAP2 is a transmembrane cell adhesion protein and its homozygous loss in humans results in severe developmental delays and intellectual disabilities that contribute to many developmental disorders such as Pitt-Hopkins Syndrome, cortical dysplasia focal epilepsy syndrome, and ASD [6]. Mice carrying a homozygous loss of CNTNAP2 (*Cntnap2* knockout [*Cntnap2* KO]) exhibit an ASD-like phenotype, including increased repetitive behaviors and deficits in sociability and communication [7]. In addition, major neuropathological features were observed in *Cntnap2* KO mice, such as deficits in neuronal migration of cortical projection neurons and a reduction in the number of striatal GABAergic interneurons [7]. We previously showed significant disruptions in DNA methylation levels in *Cntnap2* KO mouse hippocampal brain tissues on a significant number of genes associated with ASD in humans, suggesting a role for DNA methylation modulation in the pathogenesis of ASD [8].

Since we and others have found that differential DNA methylation is enriched in enhancers [9, 10], we hypothesized that the epigenome alters gene expression by influencing long-range enhancer/promoter interactions. Recent work have suggested that integration of genomic spatial conformation and DNA methylation profiles can identify a functional role between long-range chromatin interactions and the methylome in regulating gene expression [11]. Accordingly, we examined 3-dimensional protein-centric chromatin conformation (Hi-ChIP), whole genome DNA methylation, and transcriptomic patterns in hippocampal tissue from *Cntnap2* KO mice, a brain region implicated in ASD-like phenotypes [7]. Integration of these data enables us to determine whether multi-omic analysis improves the resolution of key molecular pathways contributing to the complex ASD-like phenotype in the *Cntnap2* mouse model.

## Materials and methods

### Mice

*Cntnap2* KO male and female mice [12] were purchased from the Jackson Laboratories (Bar Harbor, ME) and maintained on C57BL/6J background for more than 20 generations. Mice were housed under uniform conditions in a pathogen-free mouse facility with a 12-h light/dark cycle. Food and water were available *ad libitum*. All experiments were performed in accordance with the relevant guidelines and regulations and approved by the University of Wisconsin–Madison Institutional Animal Care and Use Committee (M005144).

### Genotyping

*Cntnap2* homozygous knockout (*Cntnap2* KO) and wild-type (WT) littermates were genotyped using the following primers: homozygous knockout Rev: CGCTTCCTCGTGCTTTACGGTAT, Common: CTGCCAGCCCAGAACTGG, WT Rev: GCCTGCTCTCA GAGACATCA. PCR amplification was performed with one cycle of 95 °C for 5 min and 35 cycles of 95 °C for 30 s, 56 °C for 30 s, and 68 °C for 30 s, followed by 68 °C for 10 min. The *Cntnap2* KO and WT alleles were obtained with 350-base pairs and 197-base pairs PCR products, respectively.

### Tissue acquisition and DNA/RNA extraction

Three-month old female *Cntnap2* KO and WT mice (*N* = 3 per group) were anesthetized with isoflurane (2 h after lights on). Mice were then perfused with 25 milliliters of 1X PBS to remove blood from the brain and the hippocampus was extracted and immediately flash-frozen in 2-methyl butane and dry ice. The whole hippocampus (∼30 milligrams of tissue) was homogenized and DNA and RNA was extracted using the AllPrep DNA/RNA mini kit following the manufacturer protocol (Qiagen, Cat. No. 80204).

### RNA library preparation, sequencing, and analysis

Approximately 300 nanograms of total RNA per sample were forwarded to Novogene Corporation Inc. for poly-A enriched library construction and sequencing. Illumina NovaSeq platforms were used for sequencing cDNA libraries. These platforms utilize a paired-end 150 bp sequencing strategy. For the RNA sequencing analysis, an average of 24 million paired-end reads were sequenced per sample. An initial check of quality was assessed for each pair-mate using FastQC. High quality, paired-end reads were trimmed for adapter contamination using trim_galore, which was run with default parameters. Trimmed and filtered reads were aligned to the Mus musculus genome (mm10) using RSEM v1.3.1 [13], which utilized STAR v2.7.0 [14]. An average of 22.3 million reads (93%) were successfully aligned for each sample. Gene expression was calculated using RSEM followed by differential expression analysis using DESeq2 V1.44.0 [15]. To account for multiple comparisons and to reduce false positives, a false discovery rate (FDR) of 0.05 was employed to identify differentially expressed genes. Comparisons to human transcriptome data used homologene R package for comparing orthologs. The NCBI taxonomy id used for human and mouse are 9606 and 10090, respectively.

### Quantitative real-time PCR

Three hundred nanograms of the RNA that was used for RNA-seq was converted to cDNA with the SuperScript™ VILO™ cDNA synthesis kit (Thermo Fisher #11754050). TaqMan real-time PCR assays were identified for select genes of interest (assay IDs: Ephb2 Mm01181017_m1; Naa38 Mm01337129_g1; Emc8 Mm00477859_m1; Synpo2 Mm03809162_m1) along with an endogenous control (Gadd45a Mm00432802_m1). 10 µL reactions were setup according to manufacturer guidelines (TaqMan Fast Advanced MasterMix for qPCR #4444557) in 96-well plates. Plates were placed on a QuantStudio™ 3 Real-time PCR System (Thermo Fisher #A28136) and cycled as follows: UNG incubation 50 °C (2 min), Enzyme activation 95 °C (20 s) followed by 40 cycles of 95 °C (1 s) and 60 °C (20 s). Result files were uploaded to the ThermoFisher Connect Platform and analyzed with the Relative Quantification application (analysis version 1.1). Relative quantification analysis setting included use of Gadd45a as the endogenous control across plates, setting WT mouse #512 as the reference sample, and WT as the reference biogroup. ΔΔCT and relative quantity (RQ) values were calculated with default settings. Significance was determined utilizing a T-Test for each target assay.

### Identification of differentially methylated regions (DMRs) and DMRs-associated genes

In the present study we used a dataset recently published by our group [8] that compared DNA methylation levels at ∼21 million CpGs in 3-month old female *Cntnap2* KO (*N* = 3) and WT (*N* = 3) mice; these were the same mice and tissues used for RNA sequencing analysis. Candidate regions were identified in dmrseq using the following parameters: minimum number of CpGs in the region set to 5 CpGs, a smoothed methylation difference of > 5% between groups, and a maximum of 10 permutations performed. Candidate methylated regions were identified and permutated, revealing a total of 3052 differentially methylated regions (DMRs, permutation *P*-value < 0.05; >5% differential). This > 5% differential in methylation level cutoff was selected based on previous epigenomic literature [16–18]. Annotation of the DMRs to standard genomic features and genes obtained from the UCSC mouse genome (mm10).

### Hi-ChIP sample preparation and data analysis

A separate cohort of three-month old female *Cntnap2* KO and WT mice (*N* = 10 per group) were anesthetized with isoflurane (2 h after lights on). Mice were then perfused with 25 milliliters of 1X PBS to remove blood from the brain and the hippocampus was extracted and immediately flash-frozen in 2-methyl butane and dry ice. Pools of 10 whole hippocampus (~350 milligram of tissue) per genotype were shipped to Dovetail Genomics^TM^ for Hi-ChIP (genome scale chromatin proximity mapping) sample preparation using antibodies against proteins linked to active transcription (RNA Polymerase II [Pol II, Active Motif, Catalog #61667] and Histone H3 Lysine 27 acetylation [H3K27ac, Cell Signaling Technology, Catalog #8173S]) [19, 20]. HiChIP library preparation: chromatin was fixed with disuccinimidyl glutarate (DSG) and formaldehyde in the nucleus. Fixed chromatin was digested in situ with micrococcal nuclease (MNase) and then extracted upon cell lysis. Chromatin fragments were incubated with the respective antibody (Pol II or H3K27ac) overnight for chromatin immunoprecipitation. The antibody-protein-DNA complex was pulled down with protein A/G-coated beads. Chromatin ends were repaired and ligated to a biotinylated bridge adapter followed by proximity ligation of adapter containing ends. After proximity ligation, crosslinks were reversed, and the DNA was purified from proteins and converted into a sequencing library. Biotin-containing fragments were isolated using streptavidin beads before PCR enrichment of the library. The sequencing library was generated using Illumina-compatible adapters. Hi-ChIP raw fastq sequence files were forwarded to Dr. Alisch’s laboratory via a secure website.

FitHiChIP (Version 11.0) [21] was used to infer significant chromatin interactions between genomic loci. FitHiChIP is a statistical tool designed to account for the systematic genomic distance bias inherent in Hi-Chip data, utilizing the spline model to penalize the physical interaction between two loci with high proximity in terms of genomic distance. This model constructs the background (or null) distribution of each pair of loci and generates *P*-value test statistics along with FDR values, which determines whether a physical interactions level is significantly higher than the background. For each genotype (*Cntnap2* KO and WT) and protein of interest (Pol II and H3K27ac), we utilized a resolution of 5 kilobase (Kb) bins and considered chromatin interactions occurring within genomic distances ranging from 5 kilobase to 1 megabase (Mb). We focused on chromatin interactions where at least one locus overlaps with ChIP-seq peaks (q-value < 0.2, Supplementary Table 1) inferred from MACS2 (Version 2.2.7.1) [22], which was applied to the protein-specific 1-dimensional Hi-ChIP data. The FDR (q-value) < 0.05 criterion was applied to both genotypes and ChIP proteins to identify significant interactions across all the chromosomes. After obtaining the sets of significant chromatin interactions from FitHiChIP for each of the four conditions (WT, Pol II; WT, H3K27ac; KO, Pol II; and KO, H3K27ac) we defined interactions as reproducible chromatin interactions for each genotype if the interactions are significantly enriched with both ChIP proteins (Pol II and H3K27ac).

### Cumulative and normalized chromatin interactions intensity

The chromatin interaction intensity for a gene was defined as the summation of contact counts of the loci overlapping with the gene on at least one locus. The intensity values of each of the four HiChIP contact matrices corresponding to a genotype (*Cntnap2* KO or WT) and a protein (Pol II or H3K27ac) were normalized by the depth of the contact matrix. Specifically, the intensity score of a gene in each of the four conditions was divided by the depth of the contact matrix corresponding to the condition, and the average depth across the four contact matrices was multiplied back to scale up. The depth is defined as the total contact count for a contact matrix. As an alternative measure of contact count strength for a gene, we defined a gene specific cumulative -log_10_FDR cumulative value, which refers to the sum of the -log_10_(q-value) values across loci overlapping the gene on at least one locus. The locus specific q-values were obtained from FitHiChIP results.

### Genomic annotation and normalization of contact counts

The two sets of reproducible chromatin interactions (WT: *N* = 7439 and *Cntnap2* KO: *N* = 8985) were annotated to genes and genomic regions using the Mus musculus genome (mm10) Gene Transfer Format (GTF) file. We annotated each locus (5 Kb bin) in the pair with a gene symbol, where the gene body location information was obtained from the GTF file (locus must overlap with the gene body). For a more refined analysis on the regulatory regions of genes, we focused on the reproducible chromatin interactions overlapping with the promoter of genes. The promoter region of a gene was defined as three 5 Kb bins: 1) a 5 Kb bin containing the TSS of the gene (TSS bin), 2) the upstream 5 Kb bin immediately contiguous to the TSS bin (upstream bin), and 3) the downstream 5 Kb bin immediately contiguous to the TSS bin (downstream bin). If the bins that compose the regulatory region are annotated to two or more genes, all genes are considered candidate genes influenced by the observed chromatin contacts. This approach may assign promoter-associated interactions to multiple genes in gene-dense regions. However, this strategy was intentionally adopted to capture all genes whose promoters overlap the interacting intervals and that could potentially be influenced by the chromatin contacts. Consequently, gene-level promoter-enhancer interaction (PEI) assignments should be interpreted as representing promoter-proximal interaction activity rather than precise enhancer–gene relationships. All 5 Kb bins in a chromatin interaction that are not annotated to the promoter are considered enhancers. To investigate the contact counts in the promoter of genes differentially expressed, the contact counts of the three 5 Kb bins (upstream, TSS, and downstream) have been summed up for each upregulated or downregulated genes. Similar to the intensity interaction normalization, we have relied on depth normalization for the aggregated (summed up) contact counts. Specifically, the aggregated contact counts of a gene in one of the four conditions (WT or *Cntnap2* KO and Pol II or H3K27ac) was divided by the depth of the condition’s contact matrix and multiplied by the average depth across four conditions for a scale-up. However, gene-level PEI intensity does not fully correct for local genomic biases such as GC content or fragment mappability. Therefore, our main analyses focused on comparative differences in PEI intensities between KO and WT rather than absolute intensity values. In addition, interaction significance metrics used in this study were derived from FitHiChIP, which accounts for major systematic biases (*e.g*., genomic distance and coverage). An overlap of reproducible chromatin interactions from WT and *Cntnap2* KO conditions identified a total of 3369 non-genotype-specific chromatin interactions (present in both WT and *Cntnap2* KO; Supplementary Fig. 1). Only genotype-specific chromatin interactions (WT-specific *N* = 4070 and *Cntnap2* KO-specific *N* = 5616; Supplementary Fig. 1) were annotated to genes and used to investigate PEI-associated genes for the study of gene-specific PEIs. When the transcriptome data was combined with PEI data, all the reproducible chromatin interactions (genotype and non-genotype-specific PEIs) were annotated to genes and employed to visualize the distribution of genes according to expression and reproducible chromatin interactions.

### Identification of gene ontological (GO) enrichments genes

The R package clusterProfiler [23] was used if the input list contains less than 1000 genes. Gene symbols were converted to their Entrez IDs using clusterProfiler and used to find significant enrichment of ‘biologic processes’ (BP). Benjamini-Hochberg (BH) was the “pAdjust” method used with a *P*-value cutoff of 0.01 and a q-value cutoff of 0.05. Only the top 10 most significant terms are shown in the figures. Cytoscape (version 3.10.1) [24] and ClueGO Plug-in (version 2.5.7) [25] was used for all the input lists containing more than 1000 genes. The gene ontology hierarchical tree in Cytoscape was set as follows: 6 minimum level and 12 maximum level GO tree interval, a minimum of 2 genes from the uploaded list found to be associated with a term, and these genes represent at least 5% of the total number of associated genes. Complete representation of all the significant gene ontology terms (biological process) was achieved by association with functional groups based on shared genes (Kappa score is used, set by default). Only groups containing significant terms (*P*-value < 0.01, Bonferroni correction) are shown and the GO Term Fusion has been used to avoid redundant terms. The most significant term of a functionally grouped network is the leading term and represents the name of the functional group in the doughnut chart. The doughnut chart shows the percentage of terms that belong to each significant functional group.

### Transcription factor binding sequence motifs

The Hypergeometric Optimization of Motif EnRichment (HOMER) [26] suite of tools was utilized test for enrichments of the known transcription factor sequence motifs in the TSS 5 kilobase bin and enhancers that participate in the chromatin interactions. Chromatin interactions linking bins among the promoter region (upstream, TSS and downstream bins) of the same gene were removed from this analysis. Chromatin interactions involving a TSS interacting with the TSS of a different gene were considered as both TSS and enhancers since they could potentially have both roles. All remaining 5 kilobase bins that participate in chromatin interactions that were not annotated to a promoter region, are considered enhancers. We used the following parameters: mm10 as the genome reference, given size of the region, and the chromatin interactions universe consisted of all the significant chromatin interactions obtained for each genotype (WT: *N* = 56,733 pair of loci, *Cntnap2* KO: *N* = 65,579 pair of loci). Enriched motifs of vertebrate transcription factors were determined using binomial testing and a q-value ≤ 0.001 was used to determine the significance of the transcription factor binding sequence motif.

### Defining the overlap of differentially methylated regions and promoter-enhancer interactions

The R package GenomicRanges [27] was utilized to identify DMRs located in the chromatin interactions. The bins that compose the promoter region (upstream bin, TSS bin, or downstream bin) and the enhancer region participating in the interaction were extended ± 2 kilobases as shown in the Supplementary Fig. 2. Genes containing chromatin interactions within any of the regulatory region bins (upstream bin, TSS bin, or downstream bin) were excluded from this analysis.

## Results

### Downregulation of ASD-related genes in *Cntnap2* KO mice

**Fig. 1:**
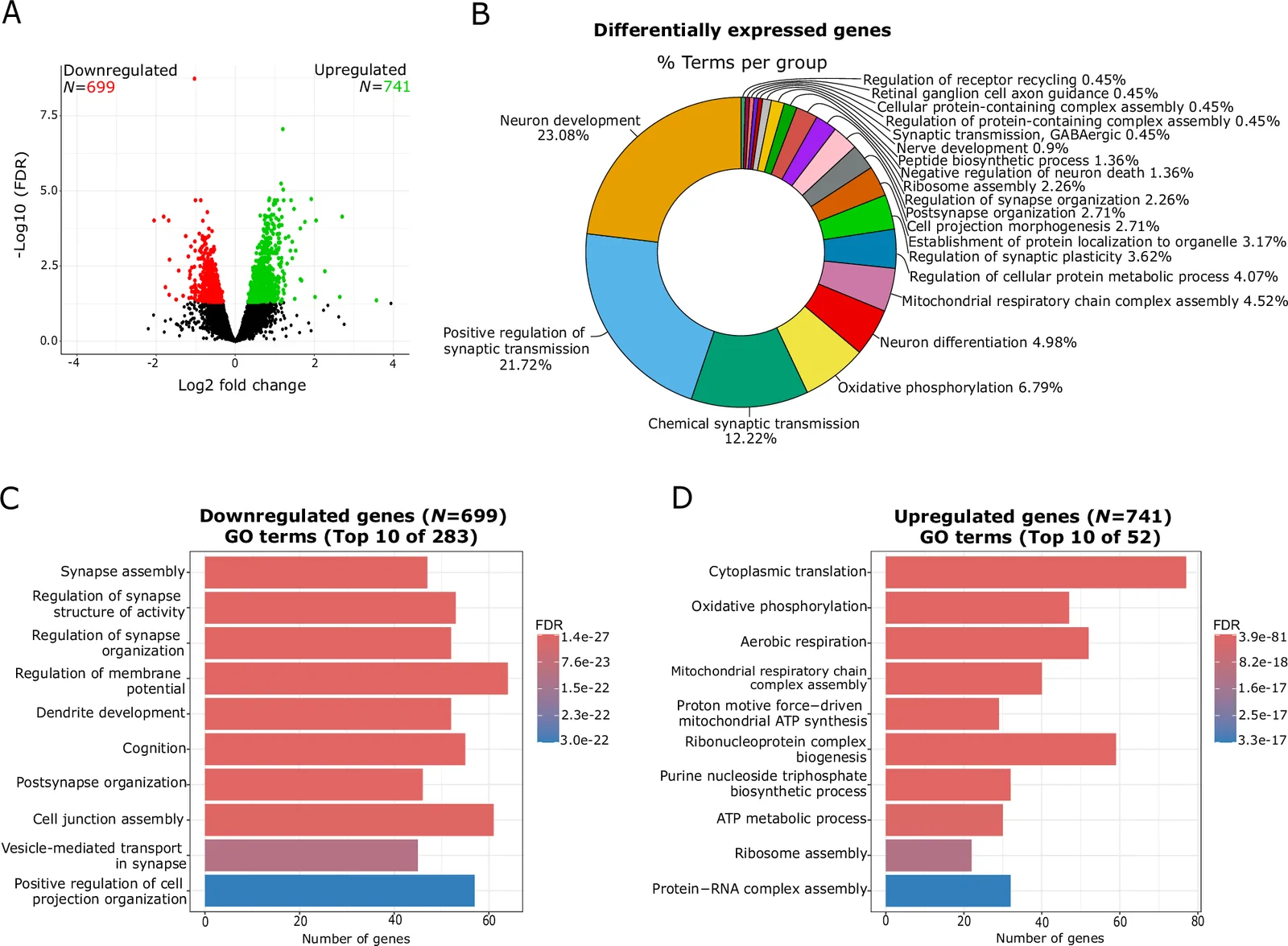
Characterization of differentially expressed genes (DEGs) between *Cntnap2* KO and WT mice. **A** Volcano plot of the transcriptomic data depicting the difference in mean (log_2_ fold change, x-axis) versus the significance (–log_10_(FDR), y-axis). Each point corresponds to a single gene. Green dots indicate upregulated genes (*N* = 741, FDR < 0.05). Red dots indicate downregulated genes (*N* = 699, FDR < 0.05). Black dots are not significant genes. **B** Doughnut chart depicting ontological pathways analysis comprising the 1440 differentially expressed genes between *Cntnap2* KO and WT mice. Gene ontology (GO) groups containing biological processes (*P*-value < 0.01, Bonferroni correction) are shown. The most significant GO term (considered the leading term) and the percentage of terms in each group compared to the total number of terms are shown. **C,** **D** Bar plot sowing the top 10 terms (y-axis) by significance (FDR < 0.05) are depicted and arranged by gene size for differentially downregulated genes (**C**) and upregulated genes (**D**) between *Cntnap2* KO and WT mice (FDR < 0.05). The number of genes linked to each GO term is shown (x-axis). The bar plot color is based on the significance (FDR, see legend).

Bulk RNA sequencing was used to determine gene expression levels in the hippocampus of adult *Cntnap2* KO and WT female mice (*N* = 3 for each genotype, Methods). To further evaluate potential sources of variation, we computed a principal component analysis (PCA) on variance-stabilized RNAseq expression values and found samples primarily cluster by genotype (Supplementary Fig. 3). Comparison of transcript levels between genotypes revealed 1440 unique differentially expressed genes, 741 upregulated and 699 downregulated in *Cntnap2* KO mice (FDR < 0.05, Fig. 1A; Supplementary Table 1 Dataset 1 & 2). The differential expression of a subset of these genes was validated using quantitative real-time PCR methodologies (*P*-value < 0.05; Supplementary Fig. 4). A pathways analysis of the 1440 differentially expressed genes identified an enrichment in neuronal ontological terms including neuron development, positive regulation of synaptic transmission, and postsynapse organization (Fig. 1B, Supplementary Table 1 Dataset 3). Independent gene ontologies (GOs) of the 741 upregulated and 699 downregulated genes showed that this enrichment in neuronal terms is determined by the downregulated genes, as the terms related to the upregulated genes comprise general cellular processes and mitochondrial functions (Fig. 1C, D, Supplementary Table 1 Dataset 4 & 5). In addition, the downregulated genes comprise a greater than 10-fold increase in the number of established ASD-associated genes (*N* = 58/699 [8.3%], SFARI database, syndromic and gene-score 1 genes), such as *Grin2a, Nrxn3*, and *Reln*, compared to the upregulated genes (*N* = 5/741 [0.7%]; Supplementary Table 1 Dataset 6). These data support that the loss of *Cntnap2* function influences the expression of biologically relevant genes contributing to the ASD-related phenotype and warrant characterization of the epigenetic mechanisms regulating these gene expression changes in *Cntnap2* KO mice.

To test the translatability of these findings to humans, we compared our data from mice to two human postmortem transcriptomic studies of prefrontal cortex and hippocampal tissues from individuals with ASD [16, 28]. These comparisons found 237 (prefrontal cortex; enrichment = 1.81, *P*-value < 2e–16) and 250 (hippocampus; enrichment = 1.17, *P*-value 0.003) differentially expressed genes shared between *Cntnap2* KO mice and humans with autism, including a three-way overlap of 86 genes (*P*-value < 2.2e–16; Supplementary Table 1, Dataset 7 & 8). These overlaps comprise several high confidence ASD-associated genes such as *SCN8A*, *NRXN3*, *STXBP1*, *NLGN2*, and *GABBR2*, suggesting convergence between mouse and human ASD-associated transcriptional changes.

### Chromatin interaction landscape in *Cntnap2* KO and WT hippocampus

**Fig. 2:**
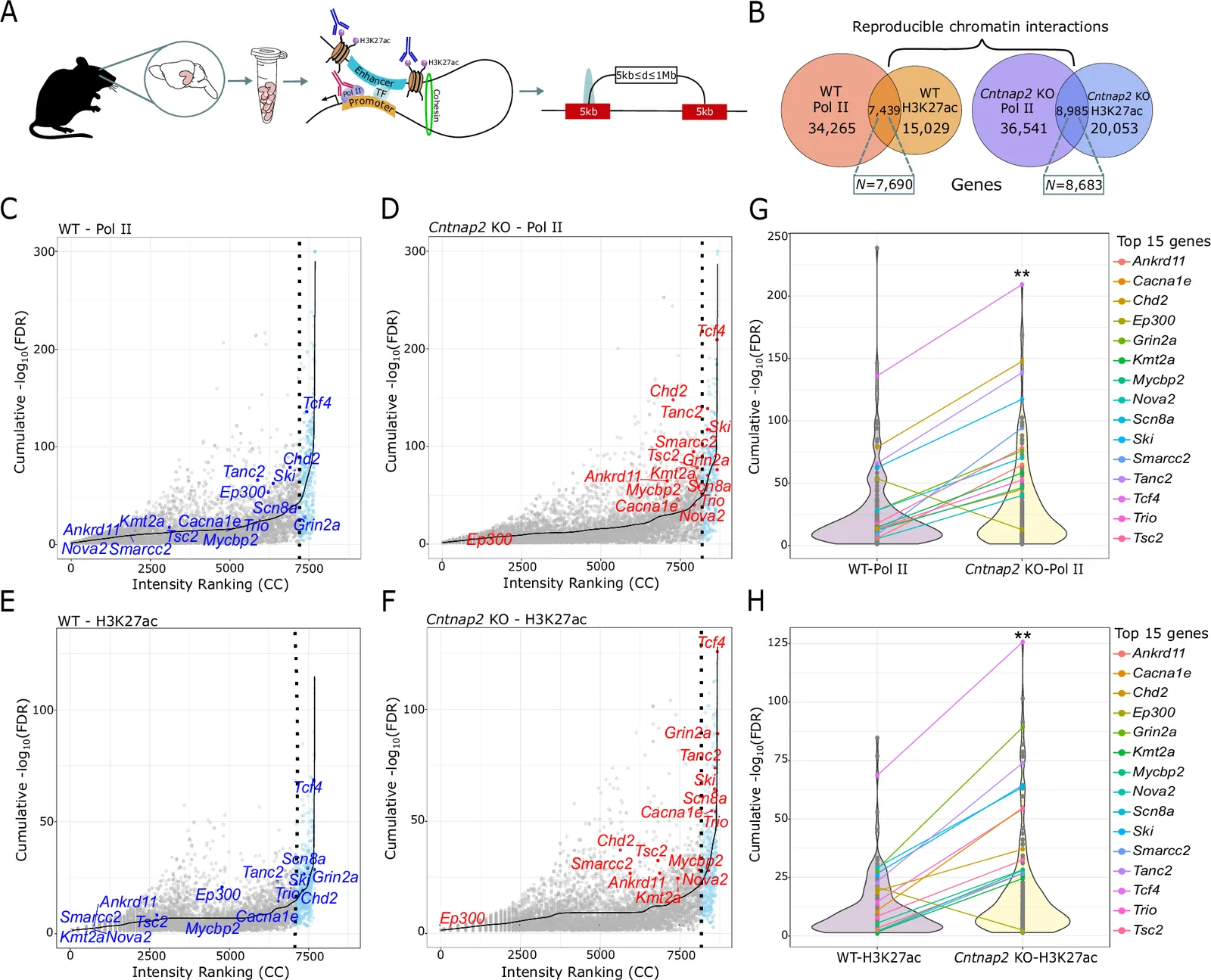
Active chromatin interactions in *Cntnap2* KO and WT mice. **A** Schematic of tissue extraction and Hi-ChIP procedure and analysis. **B** Venn diagrams displaying the number of genotype and protein-specific chromatin interactions (FitHiChIP, FDR < 0.05). The overlap in the Venn diagrams depicts reproducible chromatin interactions (set of chromatin interactions identified in both proteins [Pol II and H3K27ac] per genotype [*Cntnap2* KO and WT]). Reproducible chromatin interactions were annotated to genes (*N* = 7690, WT and *N* = 8683, *Cntnap2* KO). **C**–**F** Hockey plots of genes annotated to chromatin interactions according to gene cumulative FDR (Cumulative -log_10_[FDR], y-axis) and intensity ranking (contact counts [CC], x-axis). The y-axis was set to match genotypes for each protein (genes were excluded when meeting higher cumulative FDR than allowed for by the y-axis limit; WT-Pol II *N* = 1; WT-H3K27ac *N* = 1; *Cntnap2* KO-Pol II *N* = 14; *Cntnap2* KO -H3K27ac *N* = 4; Supplementary Fig. 8). The gross distribution of genes in terms of the significance and intensity of interactions is shown for each genotype and active transcription proteins (WT-Pol II [**C**], *Cntnap2* KO-Pol II [**D**], WT- H3K27ac [**E**], and *Cntnap2* KO-H3K27ac [**F**]). The dashed vertical line on each panel shows the elbow point of trend line curve between x-axis and y-axis on the panel, which defines the genes with high cumulative interaction scores (light-blue dots). The trend line on each panel was obtained from monotone smoothing spline. The 15 known ASD genes with the largest difference in cumulative FDR between genotypes are indicated by name on each plot (Supplementary Table 2 Dataset 9). **G,** **H** Violin plots of the 177 established ASD genes according to cumulative FDR (Cumulative -log_10_[FDR], y-axis) for each genotype (x-axis) are shown for Pol II (**G**) and H3K27 (**H**). The asterisk (**) denotes significant difference between plots (*P*-value < 0.01, Wilcoxon signed-rank test). The relative location in the plot of the 15 genes with the largest difference in cumulative FDR between genotypes are connected by a colored line and listed in the legend.

A protein-centric chromatin conformation method (Hi-ChIP) that employed separate protein immunoprecipitations with antibodies against active transcription-related proteins (RNA Polymerase II [Pol II] or Histone H3 Lysine 27 acetylation [H3K27ac]), followed by next-generation sequencing was used to determine the spatial chromatin organization in adult hippocampal tissue in the *Cntnap2* KO compared to WT mice (Fig. 2A). This approach generated an average of 217,538,116 and 258,580,420 total read counts for Pol II and for H3K27ac, respectively (Supplementary Table 2 Dataset 1). Chromatin interactions (defined as locus pairs that have at least one locus of the pair that overlaps with Chip-Seq peaks [MACS2, q-value < 0.2]) were used to identify significant chromatin interactions for each of the four conditions (WT-Pol II, *N* = 41,704; WT-H3K27ac, *N* = 22,468; *Cntnap2* KO-Pol II, *N* = 45,526; *Cntnap2* KO-H3K27ac, *N* = 29,038, FDR < 0.05, Fig. 2B, Supplementary Table 2 Dataset 1 & 2). If the interactions were significantly enriched with both ChIP proteins (Pol II and H3K27ac), we defined interactions as reproducible chromatin interactions for each genotype. *Cntnap2* KO mice had 8985 and WT mice had 7439 reproducible chromatin interactions (Fig. 2B); importantly, between-genotype chromatin interaction comparisons should be interpreted with caution (see limitations section).

Annotation of reproducible chromatin interactions to genes identified 8683 genes in *Cntnap2* KO and 7690 in WT (Fig. 2B & Supplementary Table 2 Dataset 3 & 4). The distributions of the chromatin interaction intensities of these genes were similar regardless of protein (Pol II and H3K27ac) and genotype (*Cntnap2* KO and WT; Fig. 2C–F), supporting that these findings are not biased by antibody or genotype. A subset (6–8%) of the chromatin interaction-associated genes have high chromatin interaction intensity, which defines them as super-interactive genes (WT-Pol II, *N* = 492; WT- H3K27ac, *N* = 620; *Cntnap2* KO-Pol II, *N* = 492; *Cntnap2* KO- H3K27ac, *N* = 513; Fig. 2C–F, Supplementary Table 2 Dataset 5 & 6). Comparison of chromatin interaction-associated genes to a database of high-confidence genes linked to ASD in humans (*N* = 416, SFARI database, syndromic and gene-score 1 genes) identified 235 genes in *Cntnap2* KO mice and 203 genes in WT mice, including 177 genes shared by both genotypes. These 177 ASD-related genes have more chromatin interactions and a higher intensity (based on cumulative FDR and normalized intensity) in *Cntnap2* KO compared to WT mice (*P-*value < 0.001, Wilcoxon signed-rank test; Fig. 2G, H; Supplementary Fig. 5, Supplementary Table 2 Dataset 7 & 8). These results indicate broad dysregulation of chromatin interactions among ASD-related genes, implicating a role for chromatin interactions in the observed ASD-related phenotype of the *Cntnap2* KO mice.

### Promoter-enhancer interaction (PEI)-associated genes in *Cntnap2* KO mice

**Fig. 3:**
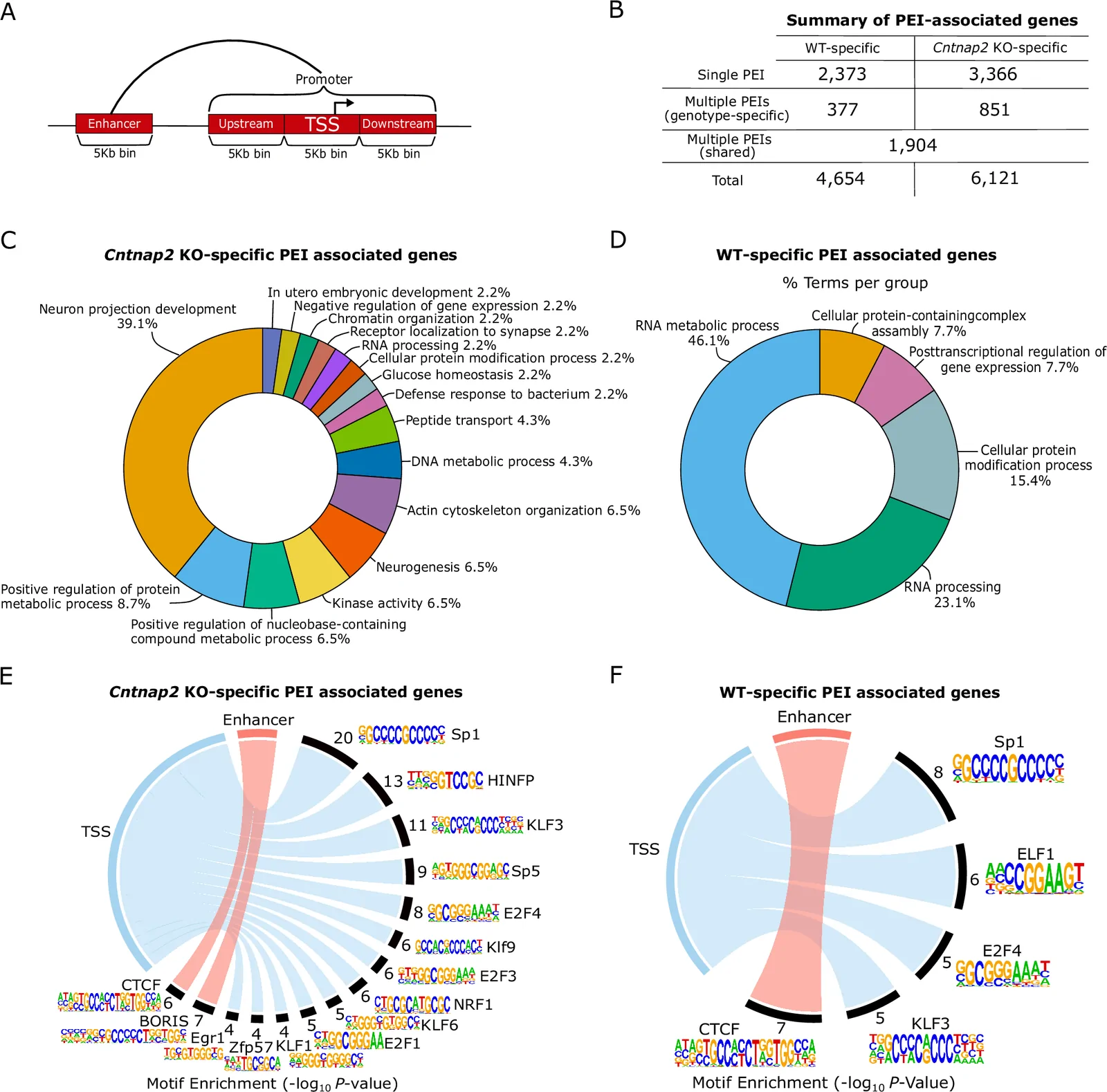
Potential functional roles of *Cntnap2* KO-specific PEI-associated genes. **A** Schematic of the definition of a promoter-enhancer interaction (PEI, Supplementary Table 2 Dataset 10). **B** PEI-associated genes according to the number of PEIs that each gene contains (single or 1 PEI, multiple or 2 or more PEIs). **C,** **D** Doughnut plots depicting ontological pathways analysis comprising the 3686 *Cntnap2* KO- (**C**) and 2452 WT- (**D**) specific PEI-associated genes. Gene ontology (GO) groups of terms containing biological processes terms (*P*-value < 0.01, Bonferroni correction) are shown. The most significant GO term (considered the leading term) and the percentage of terms in each group compared to the total number of terms are shown. **E,** **F** Circos plots of transcription factor (TF) binding sequence motif enrichments for TSS (blue) and enhancer (pink) in *Cntnap2* KO- (**E**) and WT- (**F**) specific PEI-associated genes. Arc thickness depicts TF binding sequence motif significance (-log_10_ *P*-value). Only TF binding sequence motifs with q-value < 0.0001 are shown with corresponding logo plot.

We next filtered the chromatin interaction-associated genes to those comprising a chromatin interaction in a gene promoter region, which was defined as three 5 kilobase bins flanking the transcription start site of the gene (Fig. 3A; See Methods). More promoter-enhancer interaction (PEI)-associated genes were found in *Cntnap2* KO than WT mice (*P*-value < 0.001, Fisher’s exact test; *Cntnap2* KO, *N* = 6121; WT, *N* = 4654; Supplementary Table 2 Dataset 10 & 11), which included genotype-specific genes (*Cntnap2* KO, *N* = 4217 and WT, *N* = 2750) and 1904 genes shared by both genotypes (Fig. 3B, Supplementary Table 2 Dataset 11). The majority of PEI-associated genes comprise a single genotype-specific PEI (*N* = 3366/6121 [55%] in *Cntnap2* KO and *N* = 2373/4654 [51%] in WT; Fig. 3B). GO analysis of the 4217 *Cntnap2* KO-specific PEI-associated genes identified an enrichment of terms related to nervous system development (*e.g*., neurogenesis, neuron development, and axonogenesis; Fig. 3C, Supplementary Table 2 Dataset 12). In contrast, the GO terms from the 2750 WT-specific PEI-associated genes were primarily associated with cellular metabolic processes (*e.g*., macromolecule modification, RNA metabolic process and cellular biosynthetic process; Fig. 3D, Supplementary Table 2 Dataset 12). These findings support the dysregulation of active chromatin at genes encoding proteins contributing to the altered neuronal pathways of the *Cntnap2* KO mouse.

Since syndromic forms of neurodevelopmental disorders, including ASD, involve disruptions in transcription factor (TF) function [29, 30], we next tested for enrichments of TF sequence binding motifs among the PEIs. For this analysis, we restricted the PEIs to those residing in the transcription start site 5 kilobase bin of a promoter region (Fig. 3A). The *Cntnap2* KO-specific PEIs have an enrichment of 13 TF sequence binding motifs within the promoter regions and 2 TF sequence binding motifs within the enhancer regions (q-value < 0.001). The TFs that bind to these sequence motifs are linked to neuronal processes (NRF1, E2F3, and Klf9) [31–33] and neurodevelopmental disorders, including ASD (Sp1, HINFP and Egr1; Fig. 3E; Supplementary Table 2 Dataset 13) [16, 34, 35]. In contrast, the WT PEIs have an enrichment of 4 TF sequence binding motifs within the promoter regions and 1 TF sequence binding motifs within the enhancer regions (q-value < 0.001). The TFs that bind to these WT motifs are primarily linked to processes related to cell cycle and proliferation regulation of transcription and chromatin remodeling (*i.e*., E2F4, and KLF3; Fig. 3F; Supplementary Table 2 Dataset 14) [36, 37]. Both *Cntnap2* KO- and WT-specific PEIs have enrichments of TF sequence binding motifs associated with three-dimensional chromatin architecture and gene regulation (CTCF and BORIS) [38, 39]. Together, these findings suggest that the loss of CNTNAP2 results in altered PEIs at transcription factor binding sites with known roles related to the ASD phenotype.

### *Cntnap2* KO-specific PEIs are correlated to downregulation of established ASD genes

**Fig. 4:**
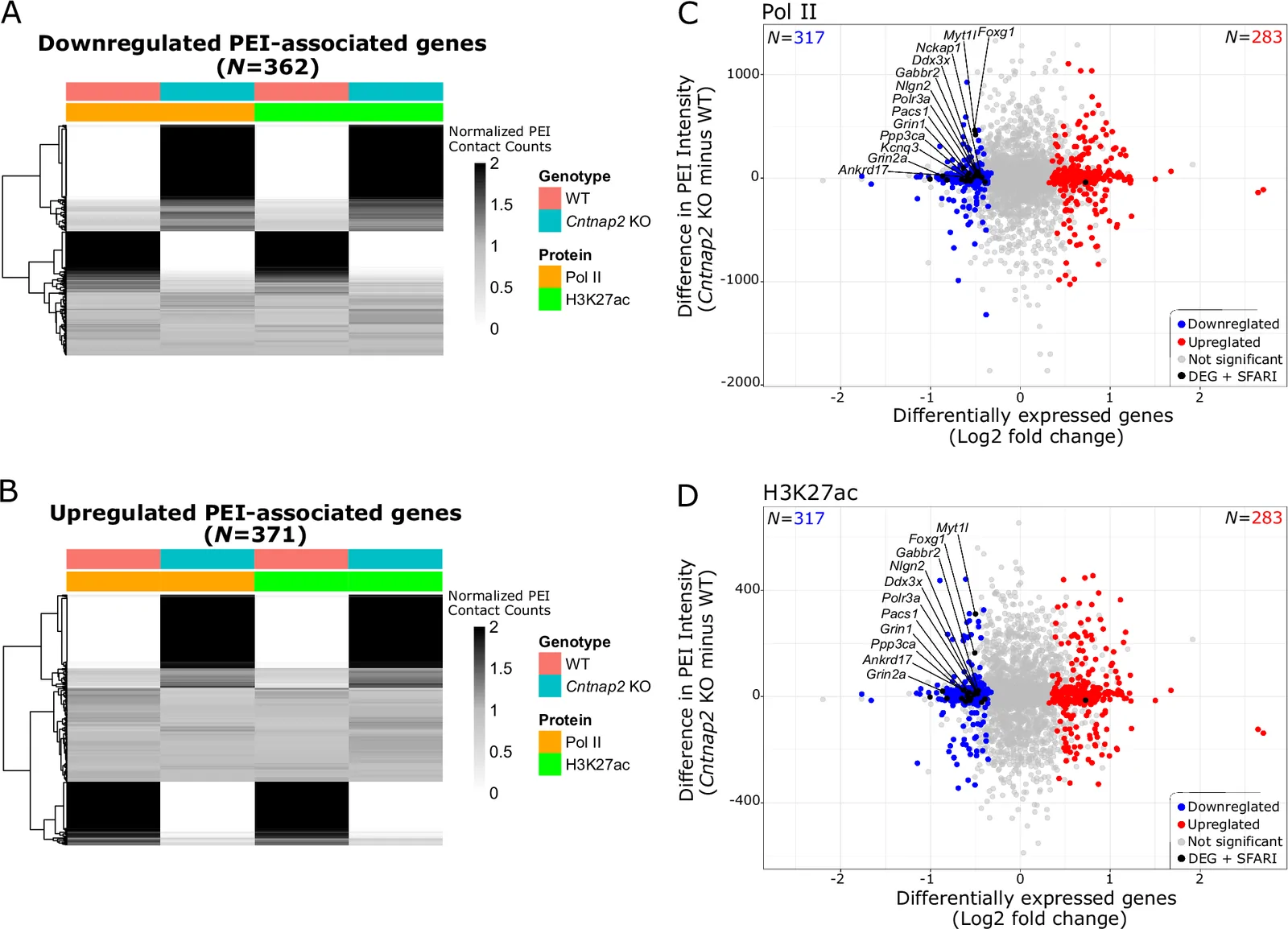
Integration of transcriptome and chromatin conformation data. **A,** **B** Heat maps of PEI-associated genes according to gene expression levels. Downregulated genes (**A**) and upregulated genes (**B**) are plotted in terms of Hi-ChIP data (normalized contact counts) for each genotype (*Cntnap2* KO [blue] and WT [pink]) and protein (Pol II [orange] and H3K27ac [green]). Each row represents a gene and the row color is based on normalized PEI contact counts. **C,** **D** Scatter plots of the transcriptome (log_2_ fold change, x-axis) and Hi-ChIP data (PEI intensity difference for each gene (*Cntnap2* KO contact counts minus WT contact counts, y-axis) for each protein (Pol II [**C**] and H3K27ac [**D**]). Downregulated (blue dots, *N* = 317) and upregulated (red dots, *N* = 283) PEI-associated genes are shown for both proteins. ASD-associated genes (SFARI database, Syndromic and gene-score 1) are indicated (black dots) and those with more PEIs that also are downregulated are called out. Grey dots indicate non-significant genes.

Integrating the transcriptome data with all PEI data independent of genotype, identified a total of 733 differentially expressed PEI-associated genes, including 362 downregulated genes and 371 upregulated genes (Fig. 4A, B, Supplementary Table 3 Dataset 1 & 2). Filtering to genotype-specific PEIs (see Methods) identified 600 differentially expressed PEI-associated genes, including 317 downregulated genes and 283 upregulated genes in *Cntnap*2 KO mice (Fig. 4C, D; Supplementary Table 3 Dataset 3). Notably, while there is not a correlation between the PEI intensity and gene expression level (*P*-value > 0.6), the majority of high confidence ASD-associated genes comprised in these data (Pol II, *N* = 13/21 and H3K27ac, *N* = 11/21) exhibit increased PEI intensity linked to reduced gene expression levels in the *Cntnap2* KO mice, including *Grin2a, Nlgn2* and *Gabbr2* (Fig. 4C, D). These finding are consistent with an enrichment of neuronal ontological terms found using the *Cntnap2* KO-specific downregulated genes (Supplementary Table 3 Dataset 4).

### Altered DNA methylation levels are correlated to differential expression

**Fig. 5:**
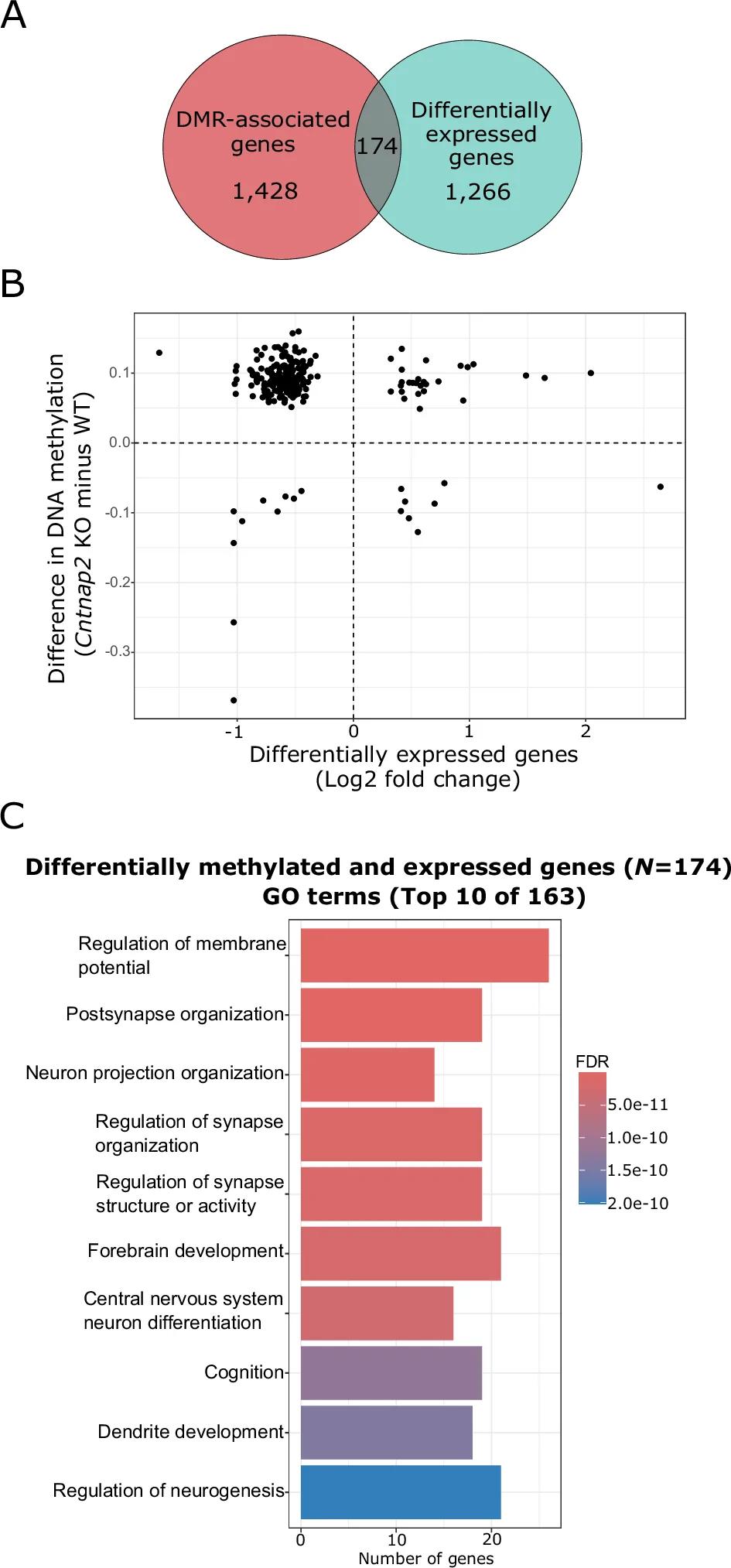
Integration of transcriptome and whole genome DNA methylation datasets. **A** Venn diagram depicts the common differentially methylated region (DMR)-associated genes (red circle, *N* = 1602) and differentially expressed genes (blue circle, *N* = 1440). **B** Dot plot of the common DMR-associated and differentially expressed genes (*N* = 166, DMR-associated genes containing both hyper- and hypomethylated DMRs [*N* = 8] were excluded) showing the association between transcriptome (log_2_ fold change, x-axis) and methylome (differential DNA methylation: average DNA methylation level in *Cntnap2* KO minus average DNA methylation level in WT, y-axis). **C** Bar plot showing the top 10 significantly overrepresented GO terms (y-axis) based on the shared DMR-associated and differentially expressed genes (FDR < 0.05). The number of genes linked to each GO term is shown (x-axis). The bar plot color is based on the significance (FDR, see legend).

Comparison of transcriptome and methylome datasets generated from the same *Cntnap2* KO and WT mice identified 174 differentially expressed genes containing differentially methylated regions (DMRs; Fig. 5A, Supplementary Table 3 Dataset 5). Seventy-eight percent of these 174 genes exhibit reduced expression and increased methylation levels in the *Cntnap2* KO mice (*N* = 130/166 genes, excluding DMR-associated genes containing both hyper- and hypomethylated DMRs [*N* = 8], Fig. 5B, Supplementary Table 3 Dataset 5). Pathway analysis of these 166 genes identified terms enriched for neurological processes, such as synapse organization, neuron differentiation, and dendrite development (Fig. 5C, Supplementary Table 3 Dataset 6). Together, these results suggest DNA methylation is modulating the expression of key genes playing a role in the observed ASD-like phenotype of the *Cntnap2* KO mouse.

### Multi-omic data integration improves the molecular resolution of the complex ASD etiology

**Fig. 6:**
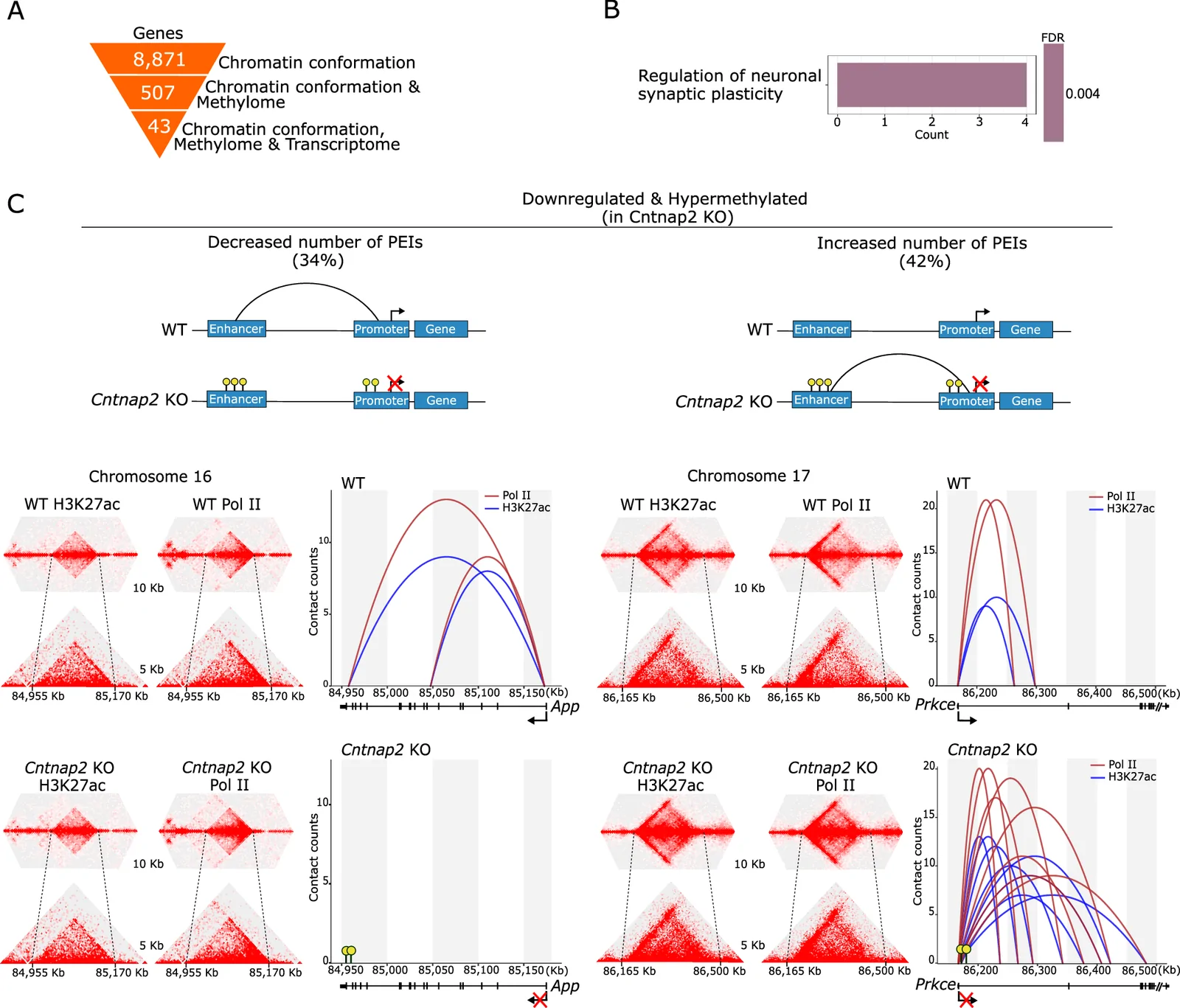
Multi-omic data integration reveals working models for the role of DNA methylation in long-range regulation of gene expression in the *Cntnap*2 KO mice model of ASD. **A** Funnel diagram showing the number of genes with disruptions in the chromatin conformation (*N* = 8871), chromatin conformation and methylome (*N* = 507) and the integration of the 3 omics (chromatin conformation, methylome and transcriptome, *N* = 43). **B** Bar plot representing the overrepresented GO term (y-axis) based on ontological pathway analysis of the 43 genes found to have disruptions in all 3 omics (*N* = 1 term, FDR < 0.05). The number of genes linked to the GO term are displayed on the x-axis. Bar color is based on FDR, see legend. **C** Gene schematics depicting the 2 most prominent scenarios comprising the 3 omics tested in *Cntnap2* KO and WT mice. Downregulated genes (crossed out arrow) with fewer PEIs (absence of black arc) and hypermethylation (yellow lollipops) in *Cntnap2* KO mice (34% of data). Contact maps and gene schematic with arc plots showing the downregulation of *App* is associated with fewer PEIs and hypermethylation in *Cntnap2* KO. A gene schematic representation of *App* and its TSS (black arrow) is shown below the arc plots. Hypermethylation is represented by yellow lollipops. Downregulation is represented with a red cross on the arrow at the TSS. Arc plots depict Pol II (red) and H3K27ac (blue) contact counts (y-axis) of all the WT- (top) or *Cntnap2* KO- (bottom) specific PEIs annotated to *App* and the genome coordinates (x-axis). In contrast, downregulated genes (crossed out arrow) with more PEIs (black arc) and hypermethylation (yellow lollipops) in *Cntnap2* KO (~42% of data). Contact maps and arc plots showing the downregulation of *Prkce* is associated with more PEIs and hypermethylation in *Cntnap2* KO. A gene schematic representation of *Prkce* and its TSS (black arrow) is shown below the arc plots. Hypermethylation is represented by yellow lollipops. Downregulation is represented with a red cross on the arrow at the TSS. Arc plots depict Pol II (red) and H3K27ac (blue) contact counts (y-axis) of all the WT- (top) or *Cntnap2* KO- (bottom) specific PEIs annotated to *Prkce* and the genome coordinates (x-axis).

Annotation of DMRs to PEIs identified 507 PEI-associated genes comprising DMRs in either the enhancer or promoter region, including 182 *Cntnap2* KO- and 276 WT-specific PEI-associated genes (Fig. 6A; Supplementary Table 3 Dataset 7). Integration of the transcriptome data to these 507 PEI-associated genes found 43 differentially expressed genes, including *App*, *Prkce*, and *Nell2*, which have roles in synapse formation and neuronal development, differentiation, and plasticity (Supplementary Table 3 Dataset 7 & 8) [40–42]. In addition, several of these 43 genes have links to neurodevelopmental disorders, including ASD (*e.g*., *Zbtb18, Cttnbp2, Gabbr2*, and *Ephb2*). Pathways analysis of the 43 genes identified one term: regulation of neuronal synaptic plasticity (Fig. 5B), suggesting that the integration of chromatin conformation, methylome, and transcriptome datasets refines the genes and pathways toward a greater resolution of the ASD molecular etiology in these data.

Forty-one of the forty-three genes could be uniquely classified by each multi-omic data set (*i.e*., upregulated or downregulated, genotype-specific chromatin conformation, and hypermethylated or hypomethylated; excluding 2 genes that contained a DMR in PEIs from both genotypes). Further characterization of these 41 genes identified that 34% (*N* = 14) were downregulated in *Cntnap2* KO mice, associated with fewer PEIs and hypermethylation (Fig. 6C; Supplementary Table 3 Dataset 8), consistent with a model in which increased methylation is associated with reduced regulatory interactions and lower gene expression. In contrast, ~42% (*N* = 17) of genes were downregulated despite exhibiting more PEIs together with hypermethylation in *Cntnap2* KO mice (Fig. 6D, Supplementary Table 3 Dataset 8), suggesting that PEIs also may function as a promoter-repressor interactions. Together, these findings support a complex context-dependent interplay between chromatin conformation and DNA methylation, where chromatin interactions and DNA methylation can act in concert or competition to regulate transcription potentially contributing to the molecular pathways linking ASD risk alleles to phenotype.

## Discussion

Novel approaches are required to overcome the challenges of characterizing genes and molecular pathways linking ASD risk alleles to phenotype. Here we tested whether the integration of epigenomic and transcriptomic data from the hippocampus of an established mouse model of ASD could improve the resolution of the complex molecular etiology of ASD. While each -omic dataset identified unique insights into the broad molecular pathogenesis of the ASD-related deficits in *Cntnap2* KO mice, the integration of transcriptome, chromatin conformation, and methylome datasets refined the gene list to 43 genes that contribute to one ontological term: regulation of neuronal synaptic plasticity. Together these data suggest the integration of multi-omic datasets provides a promising approach to reduce the heterogeneity of the complex molecular etiology of ASD.

The transcriptomic data alone offers insights into genes and pathways that are contributing to the observed ASD-like phenotype. Downregulated genes in the hippocampus of *Cntnap2* KO mice were enriched in neuronal ontological terms including regulation of synapse, dendrite development, and cognition. Upregulated genes were related to terms implicated in mitochondrial functions. These pathways are consistent with gene expression data collected from post-mortem brain tissue from persons with ASD that showed downregulated genes were enriched for ontological terms associated with synaptic function [43], mitochondrial oxidative phosphorylation, and ATP processes [44]. While we find convergence between mouse and human ASD-associated transcriptional changes, the mouse transcriptome data identified several ASD-relevant candidates that have not been reported in prior human transcriptional studies [16, 28] and are not currently classified as high-confidence ASD-risk genes. Some of these emerging candidates such as *NELL2*, *INKA2*, and *NETO1* are involved in neuronal development, dendritic spine regulation, and synaptic transmission [42, 45, 46], supporting their potential contribution to ASD-related neurodevelopmental dysfunction. We also found a 12% overlap between differentially expressed and methylated genes. The lack of a larger overlap between these datasets is consistent with previous reports [47–50] and suggests other epigenetic mechanisms, such as histone modifications and miRNAs (not evaluated here), may influence gene expression in this model. Moreover, we are reporting DNA methylation levels that may regulate gene transcription at earlier developmental time points. Further studies of other epigenetic modifications and earlier developmental time points are needed to test these hypotheses. When we investigated the overlap of the total number of differentially expressed genes (*N* = 1440) and DMR-associated genes (*N* = 1603) found here with differentially hydroxymethylated region (DhMR)-associated genes (*N* = 3554) that we reported in striatal tissue from male *Cntnap2* knockout mice [51] we found a significant overlap of genes (*N* = 62; *P*-value < 0.01; Supplementary Fig. 6). These data suggest that common correlations will be found regardless of brain regions, sex, and molecular read-out.

A key goal of this study was to test whether *Cntnap2* KO mice exhibit alterations in chromatin conformations. PEIs identify the close proximity of distal enhancers and transcriptional start sites in the three-dimensional space that regulates gene expression required to modulate important processes in the brain such as neurogenesis, neuronal development and plasticity, and memory formation [52–55]. Genetic variation in promoter and enhancer regions underscore the potential role of PEIs in neurodevelopmental disorders, including ASD [56, 57]. We not only observed PEI disruptions in *Cntnap2* KO mice but also an increased number of interactions (particularly at ASD-associated genes). The top 15 PEI-associated ASD genes have known molecular roles in neurobiology (*e.g*., calcium and sodium voltage-gated channels [*Cacna1e*, *Scn8a*], glutamate-gated ion channel [*Grin2a*], and transcription factor *Tcf4* that participates in the Wnt-β-catenin pathway) and as chromatin regulators (*e.g*., modulation of gene expression in neuronal cells [*Ankrd11*], chromatin remodeling [*Chd2*], and histone acetyltransferase [*i.e*., *EP300*]). These findings suggest that the lack of CNTNAP2 may trigger a cascade of alterations in chromatin conformation across the genome that influences the expression of genes associated with brain related processes and ASD. PEIs mediate cell type-specific functions in the brain by recruiting transcription factors (TFs) [58]. Many genes associated with ASD and neurodevelopmental disorders are transcriptional regulators able to dysregulate critical genes during development [30]. The PEIs observed in the *Cntnap2* KO mouse model may alter DNA binding of several biologically relevant TFs, such as nuclear respiratory factor 1 (NRF1). NRF1 interacts with AUTS2 and mediates the activation of genes associated with neuronal development [31]. An ASD connection to AUTS2 is consistent with studies in human cell lines showing that *CHD8*^*+/−*^ neurons (ASD model) exhibit an increase in open chromatin across the genome, with the largest chromatin change near *AUTS2* [59]. These results also suggest that different genetic variants associated with ASD may alter similar genes and pathways.

Much can be learned about the interplay of various molecular mechanisms through the study of multi-omic data sets. While increased numbers of active chromatin PEIs are typically associated with higher expression levels we observed that several ASD-associated genes were downregulated in *Cntnap2* KO mice despite exhibiting more PEIs. This evidence suggests that chromatin interactions may be competing with other regulatory mechanism that could be preventing the efficient transcription of these relevant genes. Seventy-two percent of the 43 genes obtained by integrating the multi-omic data sets exhibit hypermethylated DMRs and decreased gene expression in *Cntnap2* KO mice. A subset of these genes (32%), also comprised a decreased number of PEIs, consistent with gene regulatory models showing that increased DNA methylation levels promote chromatin compaction that reduces the chances of promoter-enhancer interactions and impedes transcription [60, 61]. Genes exhibiting decreased expression and increased number of PEIs (~40%) suggest promoter-repressor interactions, which has been shown in both fetal and adult neurons with chromatin interactions between active marks that facilitate in transcriptional repression [55]. In our model, it is not clear whether DNA methylation is a cause or consequence of these potential promoter-repressor interactions. Nonetheless, these observations highlight the context-dependent nature of chromatin interactions and underscore the need for additional functional and cell-type–dimension studies to distinguish productive from non-productive regulatory interactions in the context of ASD.

Emerging evidence suggests that although ASD is a complex heterogenous condition, it may be characterized by disruptions in common biological and molecular pathways in the brain [62, 63] Particularly, GWAS and gene expression studies have provided strong evidence that a large number of etiologically different causes in ASD have convergent dysregulated mechanisms and biological processes. Collectively, these studies emphasized disruptions in pathways associated with neuronal and synaptic function [43, 64, 65]. Consistent with these reports, the multi-omic integration of the data reported here from hippocampal tissue of a monogenic knockout mouse model of ASD reveals a refinement of the disrupted genes (*N* = 43) and molecular pathways (only one neuronal term).

Limitations should be considered when interpreting these findings. First, these presented data are from a single ASD mouse model (*Cntnap2* knockout), brain region (hippocampus), and sex (adult female), which may not capture the full molecular heterogeneity of ASD across different genetic backgrounds, brain regions, sex, or developmental stages. Future studies of additional brain regions will likely shed light into potential molecular mechanisms regionally disrupted in the ASD brain. In addition, the study of other ASD mouse models carrying monogenic gene mutations associated with different processes (synaptic plasticity, transcription factors, chromatin remodeling) are needed to test the hypothesis that distinct genetic variants associated with ASD converge on generalized molecular mechanisms that could serve as targets for early detection and the development of therapies and interventions. This study is an extension of our published findings in adult female *Cntnap2* knockout mice [8, 51]. A recent report of the prevalence of ASD among children found that girls with ASD are more likely to be classified as having an intellectual disability than boys with ASD (42.1 versus 36.9%), suggesting many autistic girls are missed or misdiagnosed [66]. Nonetheless, future studies including males, and earlier ages are imperative to determine sex-specific, and the developmental timing of the molecular pathways contributing to the ASD phenotype. Second, all three omics tested here were conducted in bulk cells that can reflect cell-intrinsic regulatory changes as well as differences in cellular composition. In the absence of single cell omics data in the mice studied here, we must rely on cell composition estimates from publicly available datasets and cell-type deconvolution algorithms. Using a publicly available mouse brain single-cell RNA-seq reference dataset [67] and the MuSiC cell-type deconvolution algorithm [68] we found that estimated cell-type fractions were largely similar between genotypes, although modest differences were observed for neurons (nominal *P*-value = 0.03) and immune cells (nominal *P*-value = 0.02; Supplementary Fig. 7). While we cannot fully exclude subtle changes in specific neuronal subtypes, our results suggest that most of the bulk RNA-seq differences reflect per-cell regulatory changes rather than shifts in cell-type composition. Third, this study generated chromatin conformation data using state-of-the-art technologies (Hi-ChIP) requiring large amounts of tissue (~350 milligram of tissue) that necessitated a separate cohort of mice, pooling 10 whole hippocampi per genotype and protein, and one technical replicate (preventing between-genotype chromatin interaction comparisons). Importantly, we only integrated Hi-ChIP data enriched in both ChIP proteins (Pol II and H3K27ac) with the transcriptome and methylome data. The integration of these three omic datasets incorporates replicate findings and excludes unique data from each omic dataset. Despite our data being consistent with large-scale transcriptomic studies of human ASD postmortem brain tissue, direct validation in human samples and functional studies will be necessary to establish causal relationships between the identified molecular pathways and ASD-related phenotypes.

In conclusion, our multi-omics approach in hippocampal tissue from a monogenic knockout mouse model of ASD improves the resolution of the complex ASD molecular etiology and aligns with studies of human ASD postmortem brain tissue. These findings offer insights into the convergent biological processes associated with the heterogeneous genetic architecture and molecular etiology of ASD.

## Supplementary information

Supplementary Figure 1

Supplementary Figure 2

Supplementary Figure 3

Supplementary Figure 4

Supplementary Figure 5

Supplementary Figure 6

Supplementary Figure 7

Supplementary Figure 8

Supplementary Table 1

Supplementary Table 2

Supplementary Table 3

## Data Availability

All data has been submitted to Gene Expression Omnibus (GEO) and is available from the corresponding author via email.
