# Supplementary material for: Multi-omic data integration improves the resolution of the molecular etiology of autism in a mouse model
Source: Mol Psychiatry. 2026 Jul 25;31(12):7196–207. doi: 10.1038/s41380-026-03772-4 (PMC13736642; doi:10.1038/s41380-026-03772-4)
Supplement: Supplementary file 1 [file 41380_2026_3772_MOESM1_ESM.pdf]

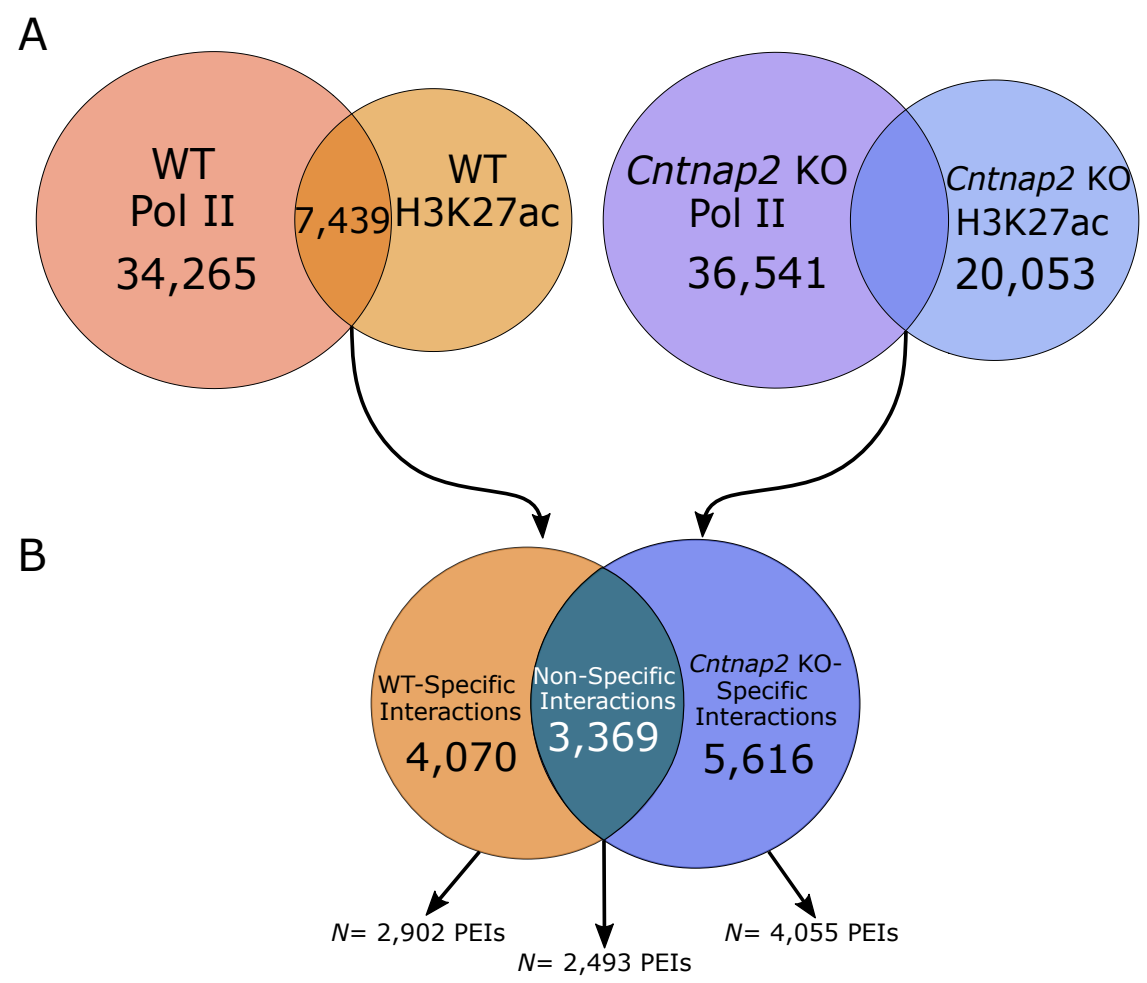

Supplementary Figure 1: Chromatin interactions in *Cntnap2* KO and WT. (A) Venn diagrams indicating the number of genotype and protein-specific chromatin interactions (FDR<0.05). The overlap depicts *N* = 7,439 WT and *N* = 8,985 *Cntnap2* KO reproducible chromatin interactions. (B) Venn diagram displaying WT-specific (*N* = 4,070) and *Cntnap2* KO-specific chromatin interactions (*N* = 5,616). The overlap in the Venn diagram depicts non-specific interactions (*N* = 3,369, present in both WT and *Cntnap2* KO). Annotation of these chromatin interactions to promoters identified WT-specific (*N*= 2,902), *Cntnap*2 KO-specific (*N* = 4,055), and non-specific (*N* = 2,493) PEIs.
