# Supplementary material for: Multi-omic data integration improves the resolution of the molecular etiology of autism in a mouse model
Source: Mol Psychiatry. 2026 Jul 25;31(12):7196–207. doi: 10.1038/s41380-026-03772-4 (PMC13736642; doi:10.1038/s41380-026-03772-4)
Supplement: Supplementary file 2 [file 41380_2026_3772_MOESM2_ESM.pdf]

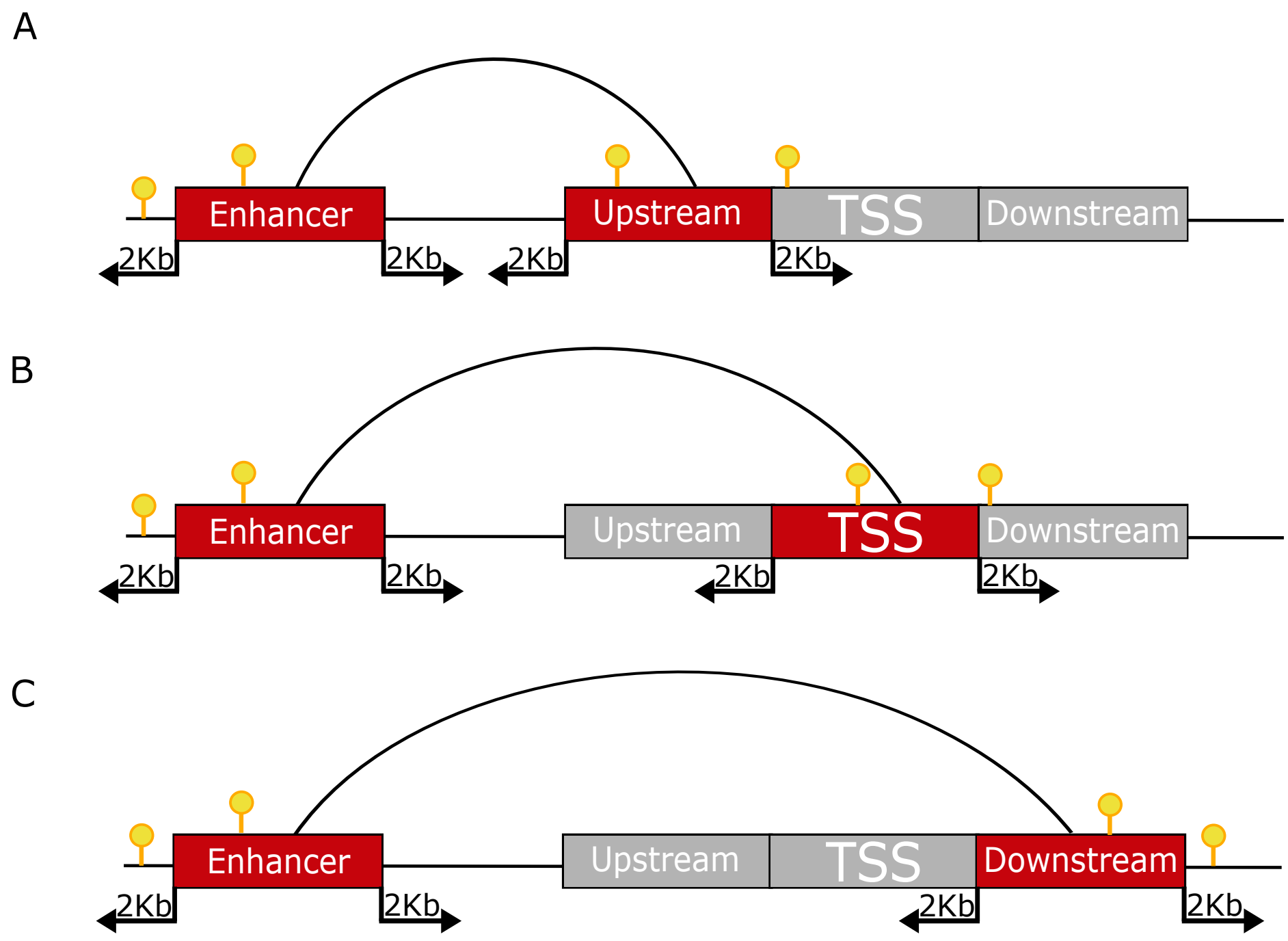

Supplementary Figure 2: Annotation of the differential methylated regions (DMRs) to Promoter-Enhancer Interactions (PEIs). The promoter region (upstream bin, TSS bin, or downstream bin) and the enhancer region were extended ± 2 kilobases (black arrows). DMRs (yellow lollipops) can reside in the enhancer region or the upstream bin (A), TSS bin (B), or downstream bin (C).
