# Supplementary material for: Multi-omic data integration improves the resolution of the molecular etiology of autism in a mouse model
Source: Mol Psychiatry. 2026 Jul 25;31(12):7196–207. doi: 10.1038/s41380-026-03772-4 (PMC13736642; doi:10.1038/s41380-026-03772-4)
Supplement: Supplementary file 3 [file 41380_2026_3772_MOESM3_ESM.pdf]

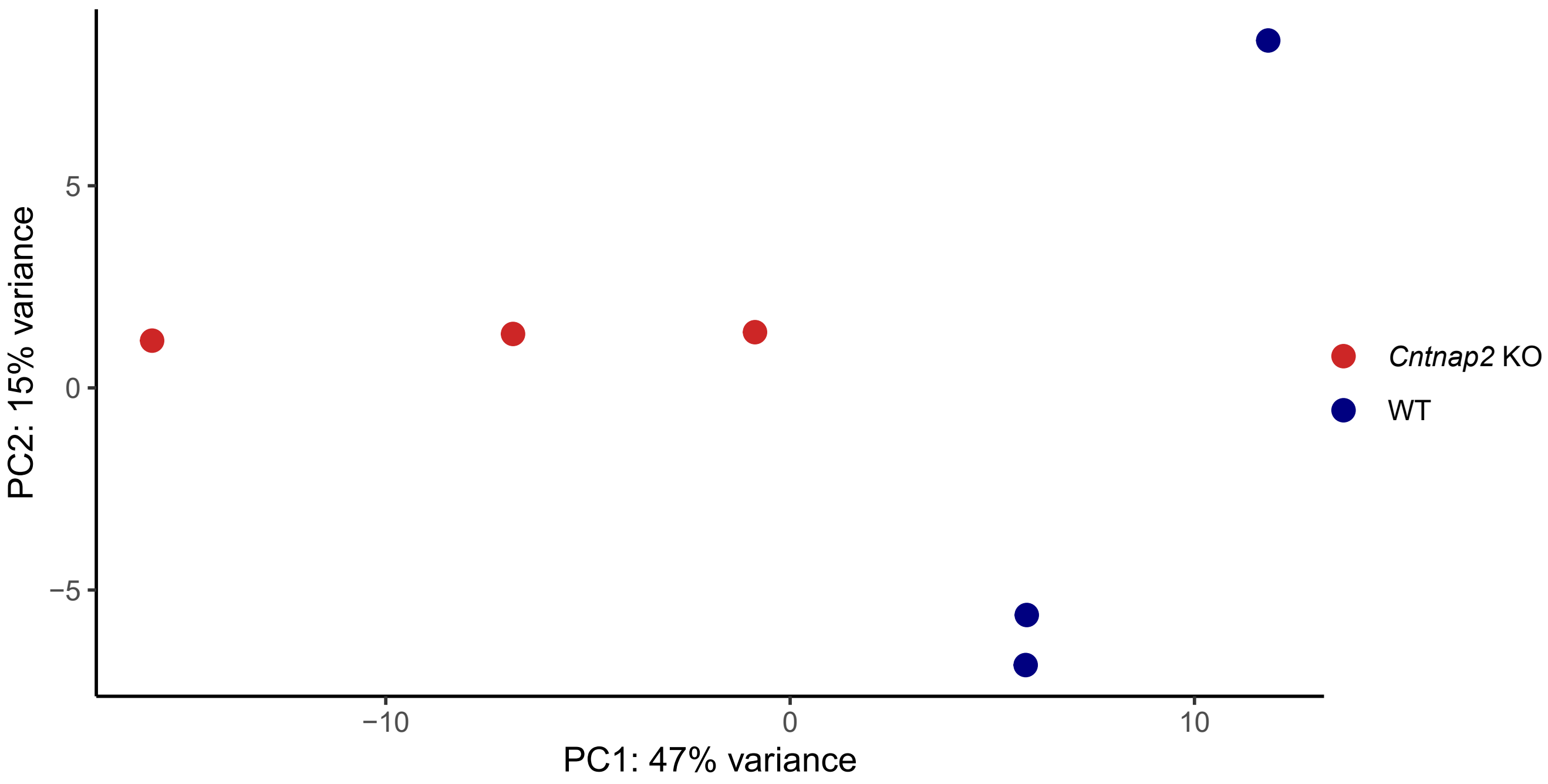

Supplementary Figure 3. Principal component analysis of hippocampal RNA-seq samples.Principal component analysis (PCA) was performed on variance-stabilized expression values from hippocampal RNA-seq samples derived from wild-type (WT) and CNTNAP2 knockout (KO) mice (n = 3 per genotype). Samples cluster by genotype and separate along PC1, which accounts for 47% of the total variance. These results indicate that genotype is the primary driver of transcriptional variation among samples.
