# Supplementary material for: Multi-omic data integration improves the resolution of the molecular etiology of autism in a mouse model
Source: Mol Psychiatry. 2026 Jul 25;31(12):7196–207. doi: 10.1038/s41380-026-03772-4 (PMC13736642; doi:10.1038/s41380-026-03772-4)
Supplement: Supplementary file 4 [file 41380_2026_3772_MOESM4_ESM.pdf]

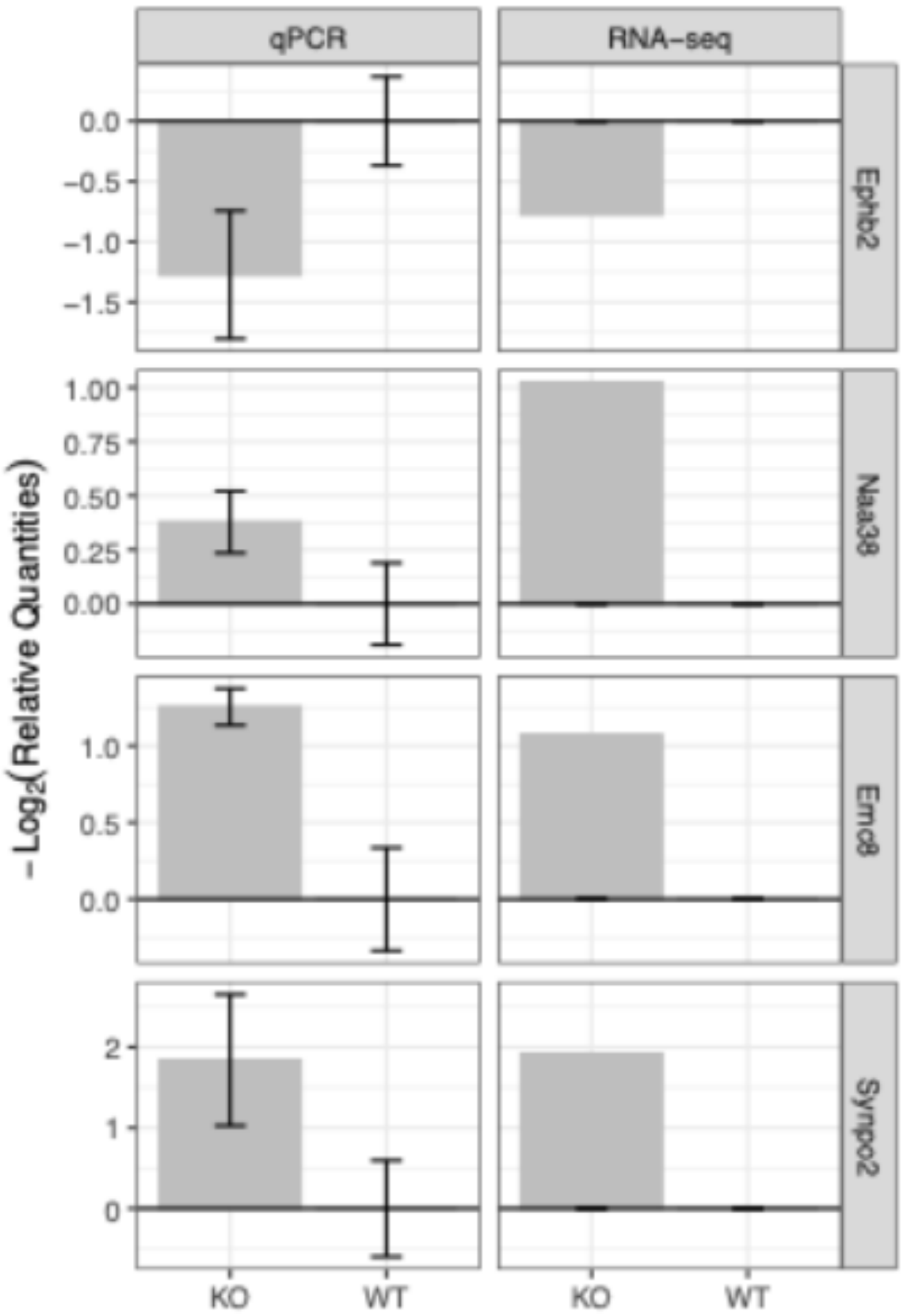

Supplementary Figure 4: qPCR validation of RNA-sequencing results. RNA extracted from mouse hippocampal tissue of both wild type and *Cntnap2*-KO was used to validate RNA-sequencing results via quantitative real-time PCR (Thermo Fisher Taqman). *Ephb2* expression levels are significantly decreased in Cntnap2-KO mice. *Naa38, Emc8,* and *Synpo2* expression levels are significantly increased in Cntnap2-KO mice. *Gadd45a* was used as the reference gene. Y-axis displays the -log2 transformed relative quantities from both the qPCR results and the RNA-sequencing results. qPCR error bars represent propagated standard deviation of ΔΔCq (fσ) from replicate measurements.
