# Supplementary material for: Multi-omic data integration improves the resolution of the molecular etiology of autism in a mouse model
Source: Mol Psychiatry. 2026 Jul 25;31(12):7196–207. doi: 10.1038/s41380-026-03772-4 (PMC13736642; doi:10.1038/s41380-026-03772-4)
Supplement: Supplementary file 5 [file 41380_2026_3772_MOESM5_ESM.pdf]

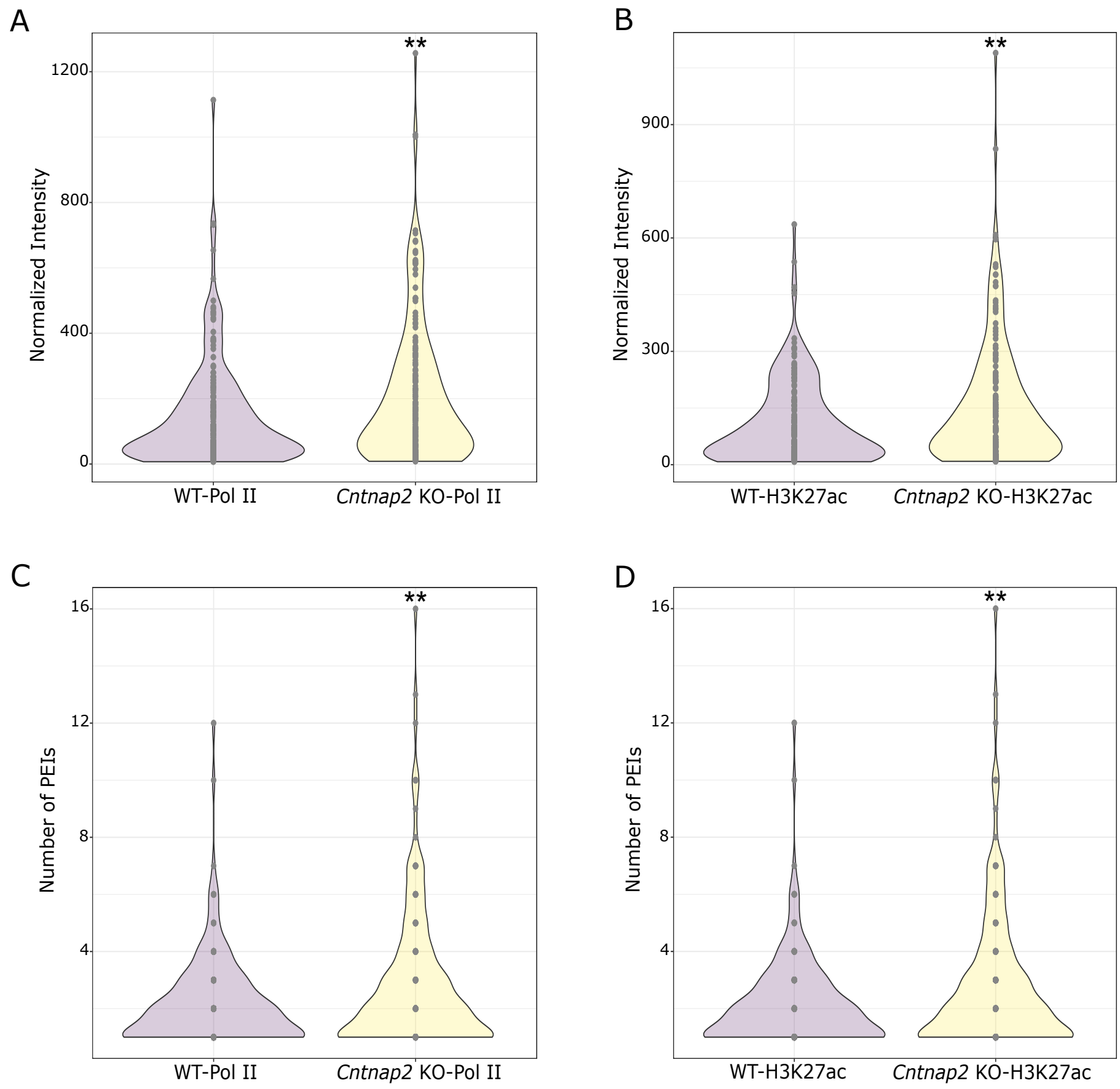

Supplementary Figure 5: Distribution of the chromatin interactions of the 177 established ASD genes. Violin plots showing the normalized intensity (y-axis, A-B) and number of PEIs (y-axis, C-D) for each genotype (x-axis) and antibody (Pol II and H3K27). The asterisk (**) denotes a significant difference between the plots (paired Wilcoxon Test, P -value <0.01).
