# Supplementary material for: Multi-omic data integration improves the resolution of the molecular etiology of autism in a mouse model
Source: Mol Psychiatry. 2026 Jul 25;31(12):7196–207. doi: 10.1038/s41380-026-03772-4 (PMC13736642; doi:10.1038/s41380-026-03772-4)
Supplement: Supplementary file 6 [file 41380_2026_3772_MOESM6_ESM.pdf]

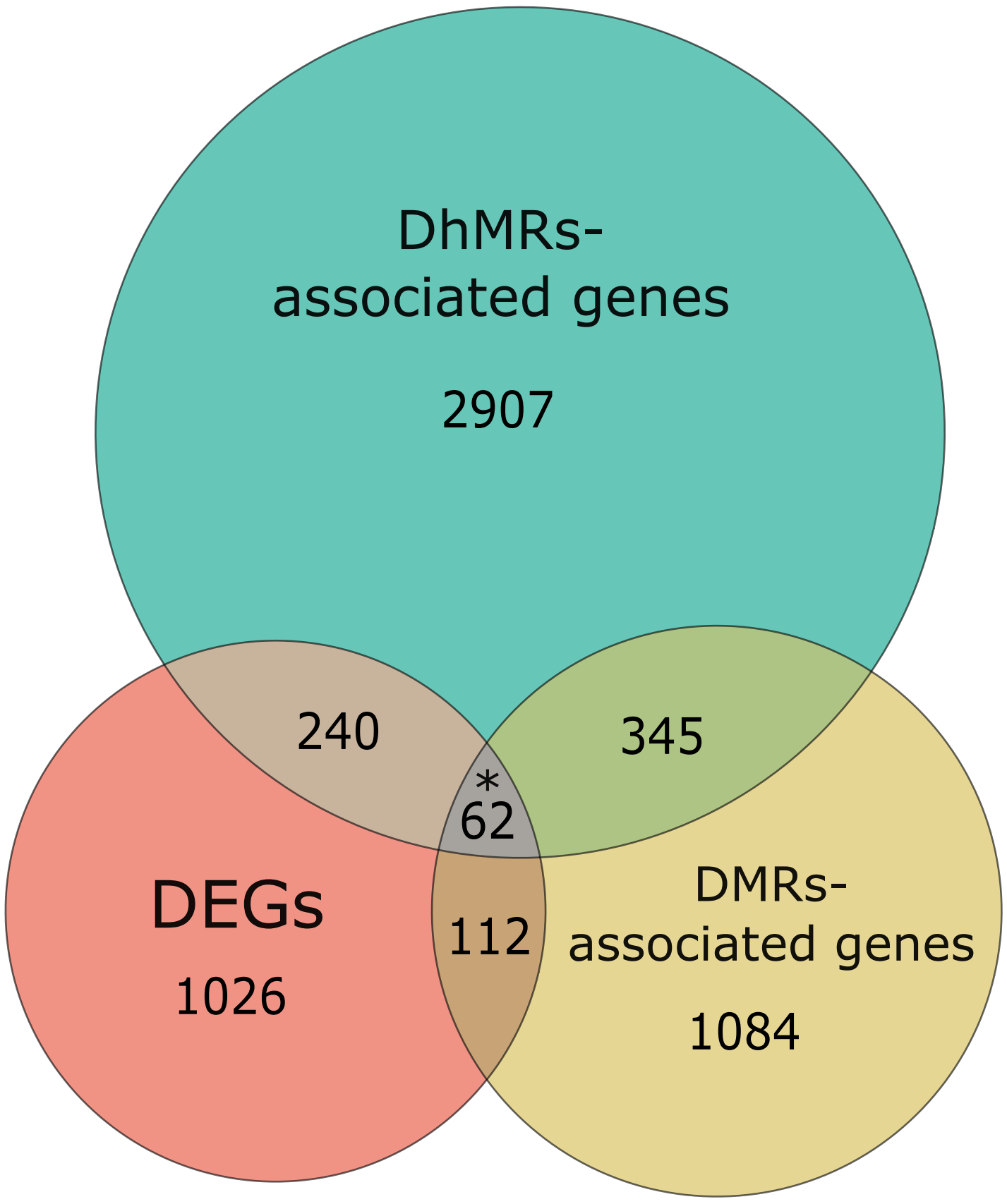

Supplementary Figure 6. Overlap between DEGs, DMRs- and DhMRs-associated genes in Cntnap2 KO mouse model of autism.The Venn diagram shows the number of genes that overlap between three datasets: differentially expressed genes (DEG, n = 1,440), differentially methylated regions-associated genes (DMR-associated genes, n = 1,603), and differential hydroxymethylated regions-associated genes (DhMR-associated genes, n = 3,554, from Papale et al., Human Molecular Genetics, 2015). Pairwise and triple overlaps are indicated, with 62 genes shared among all three datasets. *P-Value<0.01
