# Supplementary material for: Multi-omic data integration improves the resolution of the molecular etiology of autism in a mouse model
Source: Mol Psychiatry. 2026 Jul 25;31(12):7196–207. doi: 10.1038/s41380-026-03772-4 (PMC13736642; doi:10.1038/s41380-026-03772-4)
Supplement: Supplementary file 7 [file 41380_2026_3772_MOESM7_ESM.pdf]

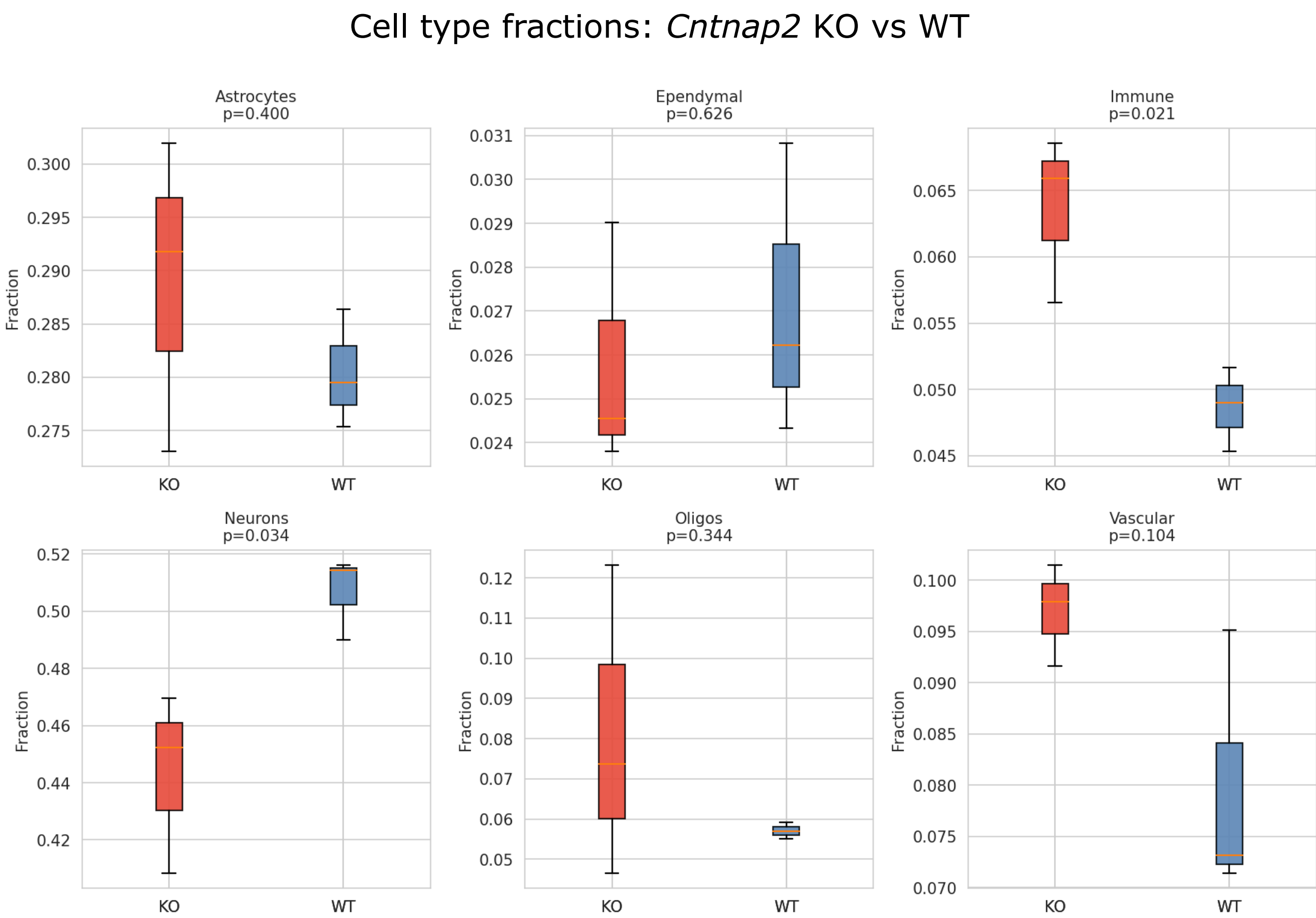

Supplementary Figure 7. Estimated hippocampal cell-type composition in *Cntnap2* KO and WT mice.Cell-type proportions were estimated from bulk RNA-seq data using the MuSiC deconvolution algorithm with a mouse single-cell reference. Bar plots show the mean estimated fraction of six major cell types—Astrocytes, Ependymal cells, Immune cells, Neurons, Oligodendrocytes, and Vascular cells—in WT (N = 3) and *Cntnap2* KO (N = 3) hippocampus. Error bars represent standard deviation across biological replicates. P-values from a two-sided t-test comparing KO and WT for each cell type are indicated above the bars. Neurons and immune cells showed nominally significant differences (P-Value = 0.034 and 0.021, respectively), while all other cell types were not significantly different (P-Value> 0.05).
