# Supplementary material for: Multi-omic data integration improves the resolution of the molecular etiology of autism in a mouse model
Source: Mol Psychiatry. 2026 Jul 25;31(12):7196–207. doi: 10.1038/s41380-026-03772-4 (PMC13736642; doi:10.1038/s41380-026-03772-4)
Supplement: Supplementary file 8 [file 41380_2026_3772_MOESM8_ESM.pdf]

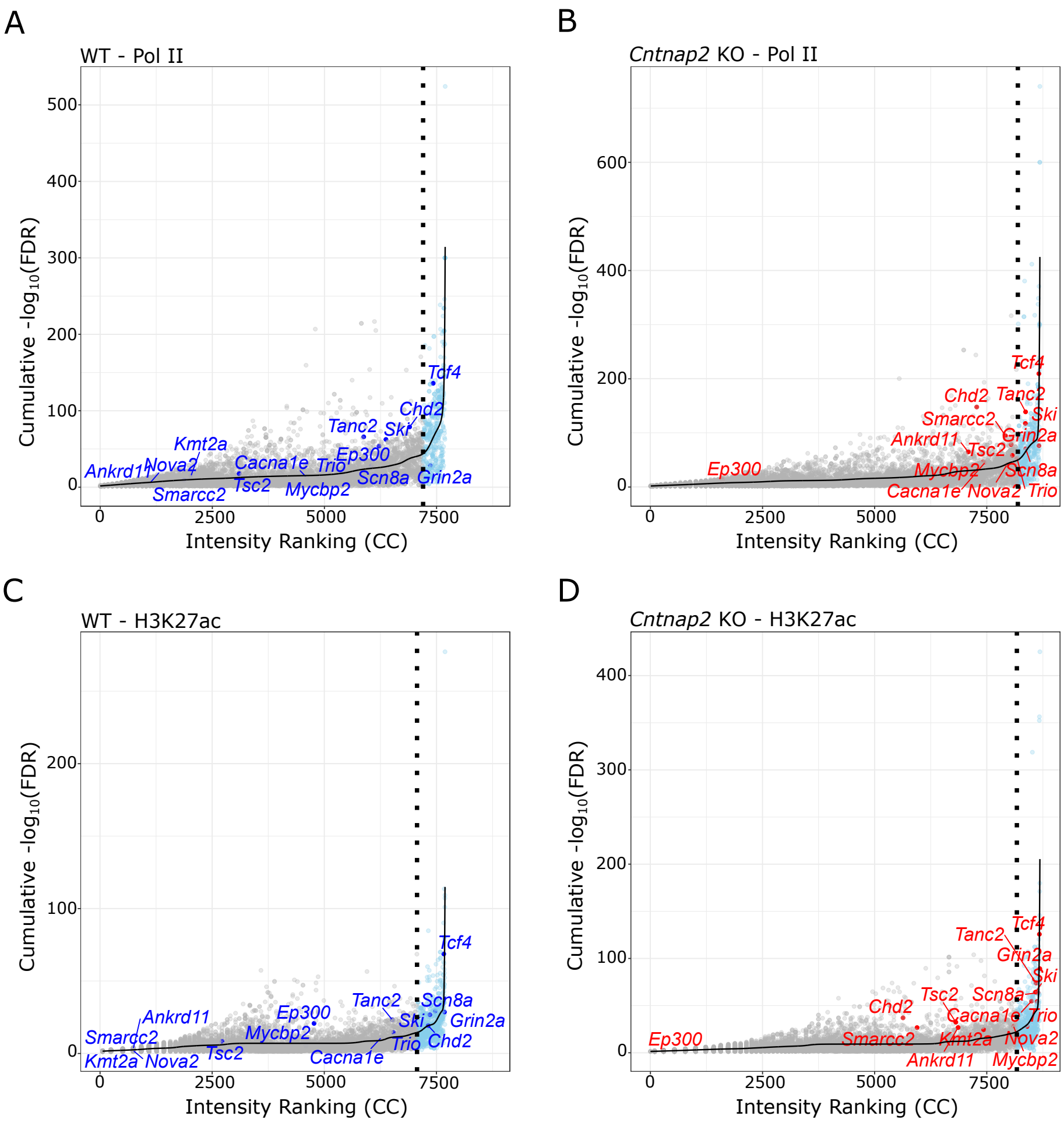

Supplementary Figure 8: The distribution of chromatin interaction. A-B) Hockey plots of genes annotated to chromatin interactions according to gene cumulative FDR (Cumulative -log10[FDR], y-axis) and intensity ranking (x-axis), depicting all the genes (including outliers). The gross distribution of genes in terms of the significance and intensity of chromatin interactions is shown for each genotype and active transcription proteins (WT-Pol II [A], *Cntnap2* KO-Pol II [B], WT- H3K27ac [C], and *Cntnap2* KO-H3K27ac [D]). The dashed vertical line on each panel shows the elbow point of trend line curve between x-axis and y-xias on the panel, which defines the genes with high cumulative interaction scores (light blue dots). The trend line on each panel was obtained from monotone smooting spline. The 15 known ASD genes with the largest difference in cumulative FDR between genotypes are indicated by name on each plot.
